# Comparative Analysis of Corneal Wound Healing: Differential Molecular Responses in Tears Following PRK, FS-LASIK, and SMILE Procedures

**DOI:** 10.3390/biomedicines12102289

**Published:** 2024-10-09

**Authors:** Dominika Janiszewska-Bil, Beniamin Oskar Grabarek, Anita Lyssek-Boroń, Aleksandra Kiełbasińska, Bernadeta Kuraszewska, Edward Wylęgała, Katarzyna Krysik

**Affiliations:** 1 Department of Ophthalmology, Trauma Centre, St. Barbara Hospital, 41-200 Sosnowiec, Poland; anitaboron3@gmail.com (A.L.-B.); kkrysik@gmail.com (K.K.); 2 Optegra Clinic in Katowice, 40-101 Katowice, Poland; 3 Collegium Medicum, WSB University, 41-300 Dabrowa Gornicza, Poland; bgrabarek7@gmail.com (B.O.G.); aleksandrakielbasinska96@gmail.com (A.K.); bkuraszewska@wsb.edu.pl (B.K.); 4 Department of Ophthalmology, Faculty of Medicine in Zabrze, Academy of Silesia, 41-800 Zabrze, Poland; 5 Clinical Department of Ophthalmology, Faculty of Medical Sciences in Zabrze, Medical University of Silesia, 40-760 Katowice, Poland; ewylegala@sum.edu.pl; 6 Department of Ophthalmology, District Railway Hospital, 40-760 Katowice, Poland

**Keywords:** myopia, tears, vision, wound healing, photorefractive keratectomy (PRK), femtosecond-assisted laser in situ keratomileusis (FS-LASIK), small-incision lenticule extraction (SMILE) procedure

## Abstract

Background/Objectives: In this study, we aimed to analyze the changes in the expression profiles of selected messenger RNAs (mRNAs) and their encoded proteins in the tears of patients undergoing photorefractive keratectomy (PRK), femtosecond-assisted laser in situ keratomileusis (FS-LASIK), and small-incision lenticule extraction (SMILE) procedures. Methods: A total of 120 patients were divided into three groups based on the laser vision correction (LVC) procedure: PRK, FS-LASIK, or SMILE. Tear samples were collected preoperatively and at 1, 7, 30, and 180 days postoperatively. The expression levels of selected messenger RNAs (mRNAs) and proteins were analyzed by using reverse transcription quantitative polymerase chain reaction (RT-qPCR) and enzyme-linked immunosorbent assay (ELISA), respectively. Results: PRK and FS-LASIK elicited significantly stronger biological responses than SMILE. Interleukin-15 (*IL-15*) expression increased notably in the PRK and FS-LASIK groups, with mRNA levels reaching fold changes of 4.65 ± 0.65 and 4.99 ± 0.28, respectively, on day 1, compared with only 2.09 ± 0.23 in the SMILE group. Vascular endothelial growth factor A (*VEGFA*) levels were also elevated in the PRK (2.98 ± 0.23 fold change) and FS-LASIK groups (3.45 ± 1.09 fold change) on day 1, while the SMILE group showed minimal fluctuations. The protein concentration analysis based on the ELISA confirmed these trends, with IL-15 levels peaking at 54.2 ± 2.5 pg/mL in the PRK group and 52.8 ± 3.1 pg/mL in the FS-LASIK group, compared with 32.4 ± 1.9 pg/mL in the SMILE group on day 1. Similarly, VEGFA protein concentrations were the highest in the PRK (72.4 ± 4.1 pg/mL) and FS-LASIK patients (69.5 ± 3.8 pg/mL) on day 1 but remained low in the SMILE patients (45.6 ± 2.3 pg/mL). By day 180, gene expression and protein levels in all groups had stabilized, returning to near-preoperative values. Conclusions: PRK and FS-LASIK induced more pronounced molecular and protein-level changes during corneal wound healing than the less invasive SMILE procedure, indicating stronger biological responses. These findings suggest that tailored postoperative care based on the specific procedure could optimize healing and patient outcomes. However, further research with larger sample sizes and longer follow-ups is needed to confirm these observations and develop personalized treatment strategies.

## 1. Introduction

Vision correction methods encompass corrective eyeglasses, hard and soft contact lenses, orthocorrection, laser vision correction (LVC), and refractive lens exchange [1,2]. The surge in popularity of LVC methods is attributed to society’s growing demands for high quality of life, extended activity in older adults, and specific occupational requirements [1,2]. LVC techniques are categorized into superficial and deep procedures, where the former encompass photorefractive keratectomy (PRK), laser subepithelial keratomileusis (LASEK), epi-Bowman keratectomy (EBK), epi-laser-assisted in situ keratomileusis (Epi-LASIK), and transepithelial photorefractive keratectomy (TE-PRK). These procedures reshape the outer layer of the corneal stroma by using an excimer laser after the corneal epithelium is removed [3,4]. Deep LVC techniques include laser-assisted in situ keratomileusis (LASIK), femtosecond-assisted laser in situ keratomileusis (FS-LASIK), and refractive lenticule extraction, such as small-incision lenticule extraction (SMILE) [5,6,7]. In PRK and LASEK, the epithelium is removed mechanically with the aid of 20% ethanol [5]. To minimize complications derived from microkeratome use, R. Kurtz and T. Juhasz introduced the use of a femtosecond laser for flap creation (the FS-LASIK method) in 2000, which significantly reduces serious flap-related complications and received FDA approval in 2001 [6,7]. The latest technique, SMILE, uses a femtosecond laser to create a lenticule in the corneal stroma, which is then extracted through a small 2–4-mm incision on the corneal surface [8,9,10].

Analyzing the variations in the cytokine expression profile in patients after laser vision correction (LVC) is crucial for several reasons [11,12,13]. First, cytokines play a pivotal role in the body’s inflammatory and healing responses, which are directly involved in the recovery process following LVC procedures [11,12,13]. Gaining insights into cytokine expression dynamics helps reveal the molecular mechanisms that drive tissue repair and regeneration in the cornea [11,12,13]. This knowledge can help optimize postoperative care, potentially reducing the hazard of complications, such as haze, infection, or prolonged inflammation [11,12,13]. Additionally, analyzing cytokine profiles may aid in identifying biomarkers for patient-specific responses to LVC, enabling personalized treatment plans that enhance outcomes and patient satisfaction [11,12,13].

Research has revealed the intricate nature of corneal wound healing, which involves various chemokines, growth factors, and cytokines [14,15,16]. During this process, the release of inflammatory mediators alters tear composition, disrupts ocular surface homeostasis, induces dry eye, and causes discomfort, significantly affecting patients’ quality of life and surgical recovery [14,15]. Hou et al. [17] showed a correlation among corneal edema, inflammation, and corneal optical density (COD), which can impact postoperative vision quality. COD is a valuable tool for pre-surgery keratoconus screening and postoperative issue monitoring, as the tracking of its changes potentially improves surgical outcomes and safety [17,18].

Therefore, the objective of this study was to evaluate the variations in the expression profiles of specific mRNAs and their corresponding proteins in the tears of patients before and after undergoing PRK, FS-LASI, and SMILE.

## 2. Materials and Methods

This study builds upon the findings and methodologies established in our previous study [19], where the surgical procedures and the postoperative period are described in detail.

This study was conducted in accordance with the guidelines of the Declaration of Helsinki and received approval from the Institutional Bioethics Committee at the Regional Medical Chamber in Krakow (approval No. 68/KBL/OIL/2020). Throughout the study, patient confidentiality and anonymity were strictly maintained. All identifying information was removed prior to database analysis. Dr. Dominika Janiszewska-Bil, PhD, MD, as an employee of Optegra Clinic and with approval from the Bioethics Committee, had access to the full patient database. The clinic consented to the use of anonymized data, which were de-identified by Dr. Janiszewska-Bil. Furthermore, Dr. Janiszewska-Bil was bound by professional confidentiality and ethical standards as a medical doctor and ensured that patient identities could not be discerned in either the database or this publication. All participants provided informed consent to participate, and patients who were unable to independently make informed decisions were excluded from this study.

The study described in this article involved the collection of tear samples from patients undergoing 3 different LVC procedures, both before surgery and at 1, 7, 30, and 180 days post-surgery. The timing of tear collection was chosen based on established corneal wound healing phases and previous research that examined cytokine and growth factor dynamics over time; these intervals capture the critical stages of the healing process after laser vision correction (LVC) procedures, including the acute inflammatory response (1 day), early wound healing (7 days), and longer-term tissue remodeling (30 and 180 days) [17,20,21].

Patients were selected based on specific inclusion and exclusion criteria (Table 1) [19]. In the next stage, we conducted bioinformatics and molecular analyses, including the use of bioinformatics databases to identify proteins involved in the wound healing process and associated with cytokine activity. This allowed us to select genes and the proteins they encode, whose expression we measured at the mRNA level by using reverse transcription quantitative polymerase chain reaction (RT-qPCR) and at the protein level by using the ELISA technique. We then performed a statistical analysis of the results obtained.

### 2.1. Study Design and Subjects

Out of 226 cases, 120 qualified for this study. A total of 14 patients were excluded from the PRK group due to moderate myopia, while 47 were excluded from the FS-LASIK group, with 35 cases of hyperopia and 12 cases of astigmatism. Similarly, 45 patients were excluded from the SMILE group, including 21 cases of hyperopia and 24 of astigmatism. The inclusion and exclusion criteria, which were consistent across all procedures, are detailed in Table 1 [19].

All patients, regardless of the LVC procedure (PRK, FS-LASIK, or SMILE), received the same postoperative medications to ensure consistency. This included a standardized regimen of topical antibiotics, corticosteroids, and lubricating eye drops. These medications were administered according to the same schedule for each patient, with no deviations in dosage or duration, unless clinically indicated due to adverse reactions or other special circumstances. Additionally, the postoperative recovery period was uniform across all groups. Follow-up appointments and assessments were conducted at the above-mentioned intervals (1, 7, 30, and 180 days post-surgery) to monitor recovery [19].

Optical coherence tomography of the anterior segment (AS-OCT; DRI OCT, Triton, Topcon, Warsaw, Poland), uncorrected distance visual acuity (UDVA), and corrected distance visual acuity (CDVA) were measured by using a Snellen Eye Chart (Hopkins Medical Products, Caledonia, MI, USA) from a 6 m distance. Intraocular pressure (IOP) was assessed with the NT-530P non-contact tonometer with pachymetry (Nidek, Poland Otical, Cieszyn, Poland) preoperatively (day 0) and postoperatively on days 1, 7, 30, and 180. These measurements provided corneal images before and after the PRK, FS-LASIK, and SMILE procedures. Detailed results of the mentioned measurements were presented in a previous publication [19].

### 2.2. Characteristics of Patients Who Qualified for PRK Procedure

A total of 20 patients (47 eyes), with an average age of 31.8 ± 5.6 years, were selected for the PRK procedure. This group consisted of 11 females (55%) and 9 males (45%), all diagnosed with mild myopia. The maximum spherical refractive error was −3.0 diopters (D), and the maximum cylindrical error was 0.5 D [19].

### 2.3. Characteristics of Patients Who Qualified for FS-LASIK Procedure

Fifty patients (92 eyes), with an average age of 39.1 ± 1.2 years, qualified for FS-LASIK surgery. The group included 34 females (68%) and 16 males (32%), all diagnosed with myopia. The maximum spherical error was −6.0 D, and the maximum cylindrical error was −5.0 D. Of these, 27 patients had mild myopia, while 23 were diagnosed with moderate myopia [19].

### 2.4. Characteristics of Patients Who Qualified for SMILE Procedure

Fifty patients (98 eyes), with an average age of 39.9 ± 2.1 years, were selected for the SMILE procedure. This group consisted of 37 females (74%) and 13 males (26%), all diagnosed with myopia. The maximum spherical error was −6.0 D, and the maximum cylindrical error was −3.5 D. Mild myopia was found in 28 patients, while moderate myopia was diagnosed in 22 patients [19].

### 2.5. Tear Collection

Tear samples were collected on the day of the operation, one week later, and one month thereafter. A 20 μL stretched micro-dropper tip (Bailey Medical Devices Co., Ltd., Taizhou, China) was carefully positioned on the lower edge of the tear river, and 20 μL of tear fluid was extracted from the conjunctival sac. Subsequently, these samples were stored in 0.2 mL Eppendorf centrifuge tubes at −80 °C to maintain their integrity. Assessments of changes in gene expression and the proteins they encode were carried out just before laser vision correction surgery and one week, one month, three months, and six months after surgery [19].

### 2.6. Molecular Assessment

#### 2.6.1. Strategy to Find Genes and Their Encoded Proteins for Molecular Analysis

Initially, 75 genes associated with the wound healing process were selected by using the PathCards Pathway Identification Database (https://pathcards.genecards.org, accessed on 14 March 2024) [22] by entering “wound healing” into the search box. To refine these results for further analysis, an over-representation test with Bonferroni correction for multiple testing was performed by using the Protein ANalysis THrough Evolutionary Relationships (PANTHER) Classification System database (https://www.pantherdb.org, accessed on 14 March 2024) [23,24]. Based on this analysis, eight genes specifically associated with “cytokine activity” in the wound healing process were selected for further study.

#### 2.6.2. Isolation of Total Ribonucleic Acid (RNA)

Total RNA isolation from tears was performed by using a Trizol reagent (Invitrogen Life Technologies, Carlsbad, CA, USA; catalog number: 15596026), following the manufacturer’s guidelines. The obtained RNA extracts were then purified by using an RNeasy Mini Kit (QIAGEN, Hilden, Germany; catalog number: 74104) and DNase I (Fermentas International Inc., Burlington, ON, Canada; catalog number: 18047019).

A qualitative assessment of the RNA extracts was conducted with 1% agarose gel electrophoresis with Simply Safe dye (EurX, Gdańsk, Poland). Electrophoretic separation was carried out by using a Submini apparatus (Kucharczyk, Warsaw, Poland). By analyzing the electropherogram under UV transilluminator light and using a computerized gel documentation system, two bands corresponding to the 28S rRNA and 18S rRNA fractions were identified. The concentration and purity of the extracts were simultaneously determined by using a spectrophotometer (Nanodrop^®^; Thermo Fisher Scientific, Waltham, MA, USA).

The purity of the RNA isolates was evaluated based on the A260/A280 absorbance ratio, which was expected to fall within the range of 1.80–2.00.

#### 2.6.3. RT-qPCR

RT-qPCR was carried out for the selected genes, utilizing glyceraldehyde 3-phosphate dehydrogenase (GAPDH) as an internal reference gene. The process was performed by using a Sensi-Fast reagent kit (Bioline, London, UK) along with the primers specified in Table 2. Both reverse transcription and PCR took place within the same tube, without modification to the reaction mixture, in a total reaction volume of 50 μL.

Each reaction was conducted in triplicate. The thermal protocol for RT-qPCR followed a standard procedure.

The results were analyzed by using the 2^−∆∆Ct^ method, where a fold change of 1 served as the control baseline, values greater than 1 indicated gene overexpression, and values less than 1 indicated gene silencing. The specificity of the RT-qPCR procedure was validated with polyacrylamide gel electrophoresis and melting curve analysis. For each biological replicate, three technical replicates were performed.

#### 2.6.4. Enzyme-Linked Immunosorbent Assay (ELISA)

The following antibodies were used for the ELISA test: anti-TGFβ1 (TGFB1 ELISA kit; MyBiosource, San Diego, CA, USA), anti-TGFβ2 (TGFB2 ELISA kit; MyBiosource, San Diego, CA, USA), anti-TGFβ3 (TGFB3 ELISA kit; MyBiosource, San Diego, CA, USA), anti-IL1B (Human IL-1 beta ELISA kit; MyBiosource, San Diego, CA, USA), anti-IL15 (IL15 ELISA Kit; MyBiosource, San Diego, CA, USA), anti-VEGFA (VEGFA ELISA Kit; MyBiosource, San Diego, CA, USA), anti-IHNB (IHNB ELISA Kit; MyBiosource, San Diego, CA, USA), anti-SLURP1 (SLURP1 ELISA Kit; MyBiosource, San Diego, CA, USA), and anti-GAPDH (Thermo Fisher Scientific, Waltham, MA, USA). All antibodies were used as recommended by the manufacturer. For each biological repeat, three technical repeats were performed.

### 2.7. Statistical Analysis

Statistical analysis was performed by using STATISTICA 13 (Statsoft, Cracow, Poland), with a threshold for statistical significance set to *p* < 0.05. The Shapiro–Wilk test was applied to determine whether the distribution of our results followed a normal distribution. Based on its results, a one-way ANOVA was conducted, preceded by checking the homogeneity of variance with Levene’s test. When the ANOVA test yielded statistically significant results, a Scheffe post hoc test was performed to identify between which observation periods the changes were statistically significant (*p* < 0.05). In turn, we used Student’s *t*-test for independent samples to compare the concentrations of selected proteins in patients before and after LVC according to gender (*p* < 0.05).

## 3. Results

### 3.1. Analysis of Transcriptional Activity of Selected Genes Based on RT-qPCR

Table 3 presents the changes in gene expression patterns over the 180-day observation period by LVC procedure. The most substantial and statistically significant changes (*p* < 0.05) in gene expression were observed for the PRK and FS-LASIK procedures during the postoperative period when compared with the day before surgery. In contrast, gene expression for the SMILE procedure remained relatively stable over the 180-day follow-up period, showing no statistically significant differences from the control (*p* > 0.05).

It should be noted that the majority of transcripts showed an increase in their transcriptional activity over time. For the PRK and FS-LASIK procedures, the silencing of gene expression was observed only for *INHBA* mRNA and *TGF-β3* mRNA on day 1 post-treatment in SMILE patients (Table 3).

### 3.2. Changes in Concentration of Selected Proteins Determined with ELISA in Patients before and after PRK, FS-LASIK, and SMILE

**Figure 1:**
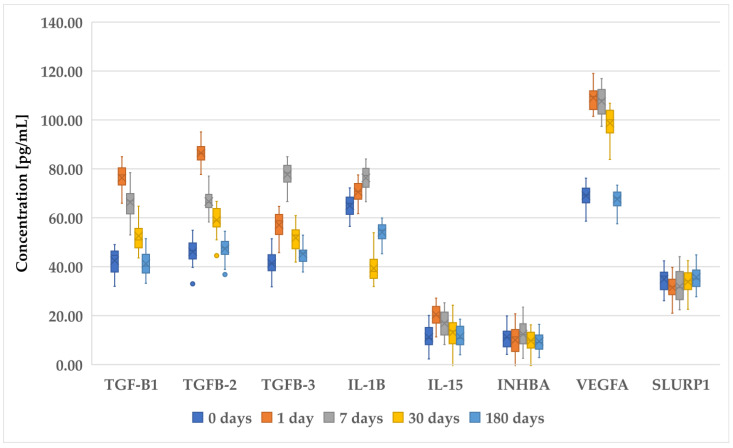
Changes in concentration profiles of analyzed proteins over a 180-day observation period in patients who underwent PRK procedure. *TGF-β1–3*, transforming growth factor beta 1–3; *IL-1β*, interleukin-1 beta; *IL-15*, interleukin-15; *IHNBA*, inhibin beta A chain; *VEGFA*, vascular endothelial growth factor A; *SLURP1*, secreted Ly-6_uPAR-related protein 1.

**Figure 2:**
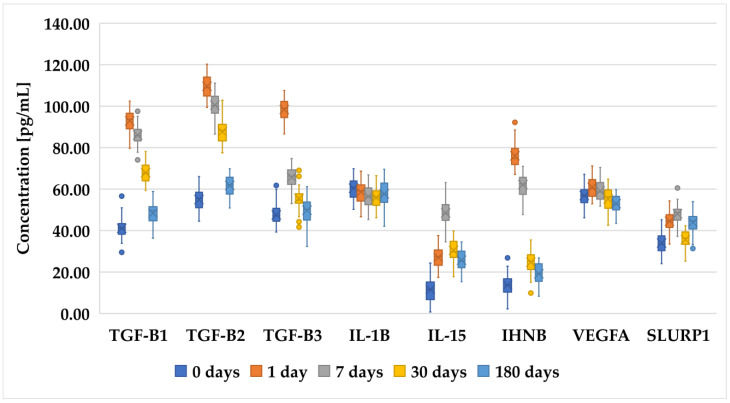
Changes in concentration profiles of analyzed proteins over a 180-day observation period in patients who underwent FS-LASIK procedure. *TGF-β1–3*, transforming growth factor beta 1–3; *IL-1β*, interleukin-1 beta; *IL-15*, interleukin-15; *IHNBA*, inhibin beta A chain; *VEGFA*, vascular endothelial growth factor A; *SLURP1*, secreted Ly-6_uPAR-related protein 1.

**Figure 3:**
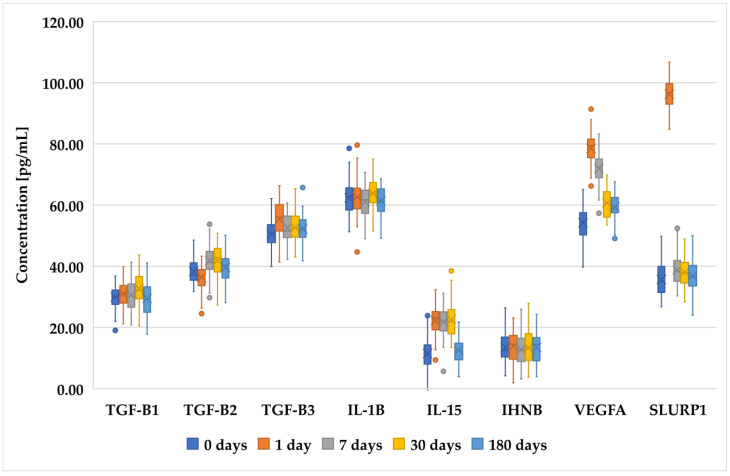
Changes in concentration profiles of analyzed proteins over a 180-day observation period in patients who underwent SMILE procedure. *TGF-β1–3*, transforming growth factor beta 1–3; *IL-1β*, interleukin-1 beta; *IL-15*, interleukin-15; *IHNBA*, inhibin beta A chain; *VEGFA*, vascular endothelial growth factor A; *SLURP1*, secreted Ly-6_uPAR-related protein 1.

Figure 1, Figure 2 and Figure 3 present an analysis of the concentrations of various proteins (TGF-β1, TGF-β2, TGF-β3, IL-1B, IL-15, INHBA, VEGFA, and SLURP1) at multiple time points (0 days (preoperative time point), 1 day, 7 days, 30 days, and 180 days) in patients who underwent PRK (Figure 1), FS-LASIK (Figure 2), and SMILE (Figure 3). In general, proteins of the TGF-β family (TGF-β1, TGF-β2, and TGF-β3) exhibited a decreasing trend, indicating a reduction in their levels over time. The IL-1β levels remain relatively low and stable across all time points, suggesting minimal fluctuation in its concentration. IL-15 showed a slight increase on day 1, followed by the stabilization of its levels. INHBA maintained a fairly consistent concentration with minor increases over time. Notably, VEGFA peaked on day 1, indicating an initial surge that gradually decreased over the observation period. Similarly, SLURP1 showed an increase on day 1, followed by stabilization thereafter.

These changes in protein concentrations may indicate an early biological response and subsequent adaptation phases, particularly for proteins such as VEGFA and SLURP1, which could be involved in the initial stages of the biological processes being studied. However, while trends were noted, not all comparisons achieved statistical significance.

The raw results of the protein and cytokine levels assessed in each patient who underwent PRK, FS-LASIK, and SMILE are presented in the Supplementary Materials (Table S1). In Table S2, we present the detailed post hoc test results of the individual comparisons of the concentration of a given cytokine at follow-up, depending on the LVC used. As a final step, we decided to assess whether the protein concentrations depended on the gender of the patients. We found statistically significant differences in SLURP1 protein concentrations between women and men only on day 7 after SMILE (49.00 ± 4.62 pg/mL vs. 46.13 ± 4.54 pg/mL; *p* = 0.04). We present the detailed results of Student’s *t*-test in Table S3.

## 4. Discussion

Over the past two decades, the increasing popularity of laser vision correction (LVC) surgery has significantly heightened interest in research focused on corneal wound healing processes. Understanding the intricate interplay of cellular and molecular mechanisms governing corneal healing has become crucial to enhancing the efficacy and safety of refractive surgical procedures. Researchers are delving into cellular responses, the roles of growth factors, cytokines, and the extracellular matrix, as well as the impact of different surgical techniques on the healing process. This comprehensive approach aims to minimize complications and optimize patient outcomes in refractive surgery [25].

However, LASIK, being a flap-based correction procedure, is associated with a higher risk of complications due to the larger area of surgical injury [26]. In contrast, SMILE, which utilizes a flapless technique, is believed to reduce the likelihood of such complications [27,28]. The biomechanical strength and stability of the cornea are better preserved in SMILE than in LASIK, where flap creation can lead to issues in the underlying stromal bed. Biomechanical studies using Bowman’s roughness index (BRI) and Corneal Speckle Distribution (CSD) suggest that tissue healing and remodeling occur more actively in SMILE than in LASIK [29].

The surgical destabilization of the cornea triggers wound healing and immune responses, which, if dysregulated, can result in postoperative complications, such as haze, ectasia, or ocular surface discomfort, including dry eye. Lie et al. and Williams et al. showed increased inflammation in LASIK compared with SMILE during the early postoperative period in rabbits and non-human primates [30,31]. Gao et al. examined ocular surface clinical parameters and tear inflammatory responses following SMILE and FS-LASIK during the early postoperative period, finding a milder ocular surface response in SMILE than in FS-LASIK. They also monitored tear inflammatory mediators from 1 day to 3 months postoperatively [21].

Despite these findings, limited information is available regarding the immediate tissue wound healing response following these refractive procedures. While biomechanical studies have highlighted differences between the two laser refractive surgeries indicative of tissue-level alterations, further investigation into cellular and wound healing responses postoperatively would enhance our current mechanistic understanding.

Epithelial wound healing occurs through several well-defined stages in a specific temporal sequence [32,33]. Studies have shown that various cytokines, such as epithelial growth factor (EGF), hepatocyte growth factor (HGF), keratinocyte growth factor (KGF), and TGF-β, play a crucial role in this process. These cytokines, due to their mitogenic properties, stimulate the proliferative activity of epithelial cells. Additionally, growth factors are present in the tear film and are often secreted by activated stromal keratocytes [34,35,36].

Thus, taking into account the fragmentary knowledge of the molecular aspects of corneal healing after LVC, in this study, we analyzed the changes in selected mRNAs and the proteins they encoded in the tears of patients who underwent one of the three LVC procedures.

Our molecular and then statistical analyses showed that more pronounced and significant changes in the expression pattern of genes and their encoded proteins associated with wound healing phenomena were observed in patients after PRK and FS-LASIK than in those patients who underwent SMILE surgery. The more invasive nature of PRK and FS-LASIK results in more significant biological responses during the healing process, evidenced by greater changes in gene expression and protein production. In contrast, the less invasive SMILE procedure results in less pronounced changes, indicating a milder healing response [37,38]. Nevertheless, it should be noted that also in patients undergoing PRK or FS-LASIK, at 180 days of follow-up, the expression profile of the selected cytokines was similar to that before surgery, indicating that the wound healing process was complete and a state of homeostasis had been reached [39].

Studies have shown that keratocyte apoptosis following corneal injury is triggered by proapoptotic cytokines released from the damaged epithelium [40,41]. Key cytokines involved in this process include interleukin (IL)-1, Fas ligand, bone morphogenetic proteins (BMPs) 2 and 4, and tumor necrosis factor alpha (TNF-α) [20,42]. These cytokines work redundantly to prevent viral resistance to the apoptotic response. For example, keratocyte apoptosis still occurred, though at lower levels, after epithelial injury in Fas-null mice [43]. Many of these cytokines are constitutively expressed by the epithelium and are rapidly released upon injury, binding to keratocyte receptors to trigger apoptosis [44].

Any form of epithelial injury can trigger this cytokine release, whether it is an epithelial scrape, a microkeratome incision during LASIK flap creation, diamond blade cuts in radial keratotomy, or even firm pressure from a contact lens. These cytokine systems likely interact in inducing keratocyte apoptosis. Notably, the IL-1/IL-1 receptor and Fas/Fas ligand systems demonstrate significant interplay. Our experiments revealed that microinjection of IL-1α into the mouse corneal stroma induced keratocyte apoptosis at the injection site. Further studies have shown that IL-1 stimulates keratocytes to produce Fas ligand mRNA and protein, whereas keratocytes typically produce Fas receptor but not Fas ligand without IL-1 induction. Thus, IL-1 stimulation results in the simultaneous production of the Fas ligand and receptor, leading to apoptosis through an “autocrine suicide” mechanism [20,45,46,47].

TGF-βs also play a crucial role in modulating the stromal wound healing response. Evidence suggests that TGF-β is involved in more aggressive wound healing, which is associated with increased stromal opacity. This highlights the importance of understanding cytokine interactions and their regulatory mechanisms in keratocyte apoptosis and wound healing to potentially improve therapeutic strategies for corneal injuries [48]. In addition, TGF-β1 facilitates the proliferation and migration of corneal stromal cells, which is essential to repopulating injured tissue. However, TGF-β1 also induces the formation of adherent myofibroblasts, which can lead to tissue fibrosis and compromise corneal transparency [49]. In contrast, Liu et al. [50] found that TGF-β1 levels remained elevated for up to one month after both procedures, but SMILE induced significantly higher levels than FS-LASIK at 24 h and 1 week postoperatively [50]. Liu et al. hypothesized that although the femtosecond laser precisely ablates the ocular surface to the corneal stroma to create a lens or flap, some energy may escape during the ablation process, causing slight epithelial damage and triggering the release of cytokines and growth factors [50]. Since SMILE involves two femtosecond laser scans compared with just one for FS-LASIK, it is plausible that the epithelium is more affected in SMILE, leading to increased TGF-β1 production. Additionally, as observed in rabbits, the lack of a Bowman’s layer in their cornea, unlike the human cornea, results in a weaker barrier between the epithelium and the stroma [51].

In our study, we observed significantly higher IL-15 expression in patients after LVC, with elevated levels of this cytokine in tears after PRK and FS-LASIK compared with SMILE. This observation was based on both RT-qPCR data, which measured mRNA expression, and ELISA data, which measured the actual protein concentrations in tears.

While the RT-qPCR results indicate higher IL-15 mRNA expression in the PRK and FS-LASIK groups, the ELISA results reveal a more complex picture. As shown in Figure 1, the IL-15 protein levels in the SMILE group (Group C) were not consistently lower than in the PRK group (Group A) at all time points. This discrepancy between mRNA and protein expression might be explained by post-transcriptional regulatory mechanisms that influence protein synthesis, stability, or degradation, as discussed previously. Additionally, it is possible that variations in local tissue responses, cytokine secretion dynamics, and protein clearance could contribute to the differences observed between the mRNA and protein levels.

It is important to note that mRNA expression levels measured by RT-qPCR provide insights into the gene’s transcriptional activity, while ELISA results reflect the functional output at the protein level. Thus, the higher IL-15 mRNA levels in the PRK and FS-LASIK groups suggest increased transcriptional activity, but this does not always translate directly into proportionally higher protein levels due to the complexity of the post-transcriptional and translational processes involved [52].

IL-15 plays a crucial role in the process of wound healing, acting as a multifaceted mediator in tissue repair that enhances the migration and proliferation of keratinocytes and fibroblasts, key players in re-epithelialization and extracellular matrix formation [53]. Moreover, Wang et al. demonstrated that IL-15 boosted IGF-1 production by dendritic epidermal T cells, thereby promoting the repair of diabetic wounds. This finding suggests that IL-15 could be a promising therapeutic agent for managing diabetic wound healing [54]. The observations made by Wong et al. indicate that IL-15 stimulates keratinocyte and fibroblast growth during the wound healing process [55]. In turn, Jones et al. suggested that IL-15 plays several complex roles in chronic wound healing. Increased levels of IL-15Rα were found in non-healing chronic wounds compared with those that are healing. Although treating keratinocytes with recombinant human IL-15 (rhIL-15) showed an increase in pro-healing traits, such as growth and migration, these effects were not statistically significant. Further research is needed to establish expression profiles in larger wound healing cohorts that include normal skin, as well as acute and chronic wound subtypes [56]. Our molecular analysis confirmed that during the wound healing process, changes in the expression of VEGFA were observed. However, to the best of the authors’ knowledge, no studies of changes in the expression profile of VEGFA in patients after LVC have been conducted to date. Nevertheless, given the biological role of VEGFA, it appears that by promoting endothelial cell proliferation, migration, and new capillary formation, this gene enhances the delivery of necessary components for tissue repair and regeneration. Additionally, VEGF aids in increasing vascular permeability, allowing for better infiltration of immune cells to the wound site, which is crucial to clearing infections and debris [57,58,59]. Interestingly, despite the inclusion of IHNHBA and SLURP1 in wound healing based on gene ontologies, their expression was not reported to be significantly affected by LVC treatments during the guided observation.

The differences observed between the RT-qPCR and ELISA results for INHBA and SLURP1 expression can be attributed to the complex nature of gene expression regulation. While RT-qPCR measures the mRNA levels, which represent the transcriptional activity of the genes, ELISA was used to quantify the actual protein concentrations in tears. The observed trends in mRNA expression might not always directly correspond to protein levels due to post-transcriptional, translational, and post-translational modifications, which can affect the final protein abundance [52].

For INHBA, while the mRNA expression levels showed a relatively consistent trend across the three surgical methods, the protein levels detected by ELISA in Group B (FS-LASIK) exhibited an increase during certain periods. This discrepancy could be due to variations in mRNA stability, differences in translation efficiency, or delayed protein synthesis. In some cases, protein accumulation may occur on a different timeline compared with the mRNA expression, leading to increased protein levels at specific time points, despite relatively stable or lower mRNA levels [60].

Similarly, for SLURP1, although the RT-qPCR results suggest a uniform trend across all groups, the protein expression in Group C (SMILE) showed an increase during certain periods. This could be explained by differential post-transcriptional regulation mechanisms, such as mRNA splicing, degradation rates, or variations in the translation process that are not reflected in the mRNA data [61]. Additionally, tissue-specific factors, the microenvironment of the surgical site, and local protein degradation dynamics could have further contributed to the observed differences [62,63].

In this study, we observed a statistically significant difference in the concentration of SLURP1 between male and female patients on day 7 after undergoing the SMILE procedure, with the latter showing higher concentrations than the former (49.00 ± 4.62 pg/mL vs. 46.13 ± 4.54 pg/mL; *p* = 0.04). Although the difference in SLURP1 expression is subtle, it highlights a potential gender-based variation in molecular responses to corneal wound healing. While IL-1β and IL-15 did not exhibit significant differences based on gender, the observed difference in SLURP1 could have implications for understanding gender-specific recovery processes after laser vision correction surgeries. SLURP1 is known for its role in modulating immune responses and epithelial cell proliferation, both of which are critical in wound healing [64]. The elevated concentration in females might indicate a more robust or faster wound healing response in the early postoperative period, which could be linked to hormonal or other biological factors that influence immune regulation. Previous studies on wound healing have shown that various factors, including sex hormones, can affect tissue repair and inflammation, which may explain this discrepancy. This finding, while requiring further validation in larger cohorts, underscores the importance of considering gender as a biological variable in postoperative care and recovery [65,66]. It also suggests the potential need for more personalized treatment strategies that account for gender-specific responses to laser vision correction surgeries. Future research should investigate the underlying mechanisms driving this difference and explore whether it translates into meaningful clinical outcomes, such as variations in healing time or complication rates between male and female patients.

These findings underscore the importance of examining both mRNA and protein levels, as each provides complementary insights into the biological processes occurring post-surgery. Future studies could explore these regulatory mechanisms in greater depth to clarify the relationship between gene expression and protein production in the context of wound healing after different LVC procedures.

Despite providing valuable insights, the study had several limitations. Firstly, the sample size was relatively small, which may limit the generalizability of the findings. Additionally, the exclusion of certain patient groups, such as those with hyperopia and astigmatism, might have influenced the results and their applicability to a broader population. Furthermore, the study’s observational period of 180 days may have not fully captured long-term changes and outcomes in cytokine expression and wound healing processes. The lack of comparative analysis with normal skin and other wound subtypes also limits the understanding of cytokine dynamics in different healing contexts. Finally, while, in this study, we focused on selected mRNAs and proteins, a more comprehensive profiling of other molecular markers could provide a fuller picture of the wound healing mechanisms following LVC procedures. Further research with larger, more diverse cohorts and extended follow-up periods is necessary to validate these findings and explore additional biomarkers.

## 5. Conclusions

This study provides valuable insights into the differential molecular responses in corneal wound healing based on the analysis of the expression profiles of selected mRNAs and their encoded proteins in tears from patients who underwent a PRK, FS-LASIK, or SMILE procedure. The results demonstrate that PRK and FS-LASIK elicited more significant changes in gene expression and protein production related to wound healing, reflecting their more invasive nature compared with the SMILE procedure. These biological responses underscore the need for personalized postoperative care to optimize patient recovery and outcomes.

While the findings contribute to a better understanding of corneal wound healing processes, the study’s limitations—such as a relatively small sample size and the exclusion of certain patient demographics—highlight the need for further research. Future studies should include larger cohorts, longer observation periods, and broader inclusion criteria to fully elucidate the molecular mechanisms underlying wound healing after laser vision correction. Addressing these limitations could pave the way for the development of more tailored treatment strategies, improving both the efficacy and safety of these procedures.

## Figures and Tables

**Table 1:** Eligibility criteria for study participants in laser vision correction surgery trials.

Inclusion Criteria	Exclusion Criteria
Voluntary informed consent to participate in the study	Lack of informed voluntary consent
Aged 18 or older	Aged under 18
Stable refraction within the year preceding the study	Opaque optical media
Myopia ≤ −6.0 D for femtoLASIK and ReLeX SMILE or ≤−2.0 D for PRK	Current and past uveitis
Astigmatism ≤ 5.0 Dcyl	History of eye injuries
CDVA ≥ 0.5 on the Snellen chart	Previous corneal laser treatment
Corneal epithelial thickness (CET) ≥ 490 µm	Previous eye surgery
Residual stromal thickness (RST) ≥ 250 µm	Autoimmune diseases
Normal corneal topography	Diabetes
	Dry eye syndrome
	Pregnancy or breastfeeding

RST, residual stromal thickness; SMILE, refractive lenticule extraction with small incision; FS-LASIK, Femtosecond-assisted laser in situ keratomileusis; PRK, photorefractive keratectomy; D, dioptric; CDVA, corrected distance visual acuity; CET, corneal epithelial thickness.

**Table 2:** Primer sequences used in RT-qPCR reaction.

mRNA	Primer Sequences
*TGF-β1*	Forward: 5′-GGCCAGATCCTGTCCAAGC-3′ Reverse 5′-GTGGGTTTCCACCATTAGCAC-3′
*TGF-β2*	Forward: 5′-CAGCACACTCGATATGGACCA-3′ Reverse 5′-CCTCGGGCTCAGGATAGTCT-3′
*TGF-β3*	Forward: 5′-CTGGATTGTGGTTCCATGCA-3′ Reverse 5′-TCCCCGAATGCCTCACAT-3′
*IL-1β*	Forward: 5′-TTTAGGATTTGGATTTTTGTTTTTT-3′ Reverse 5′- ACATCTTCCTCAACTTATCCATAAC-3′
*IL-15*	Forward: 5′-GGTTTTATTGATTTTAAGAATGAAGT-3′ Reverse 5′-ATAACAAAAAATTTCCAACAACCAT-3′
*INHBA*	Forward: 5′-AAATATGTTGTATTTGAAGAAGAGATT-3′ Reverse 5′-TTTAAAAAAAACCAAACTTCTACAC-3′
*VEGFA*	Forward: 5′-GATAGGGGTAAAGTGAGTGATTTGT-3′ Reverse 5′-AAAACAACCCAAAAATTAAAC-3′
*SLURP1*	Forward: 5′-TTTGTTTTAGTTTTTGTGTGGTTAT-3′ Reverse 5′-AAAAAATACCTAAAAAACACCTTCC-3′
*GAPDH*	Forward: 5′-GGTGAAGGTCGGAGTCAACGGA-3′ Reverse 5′-GAGGGATCTCGCTCCTGGAAGA-3′

*GAPDH*, 3-phosphoglyceraldehyde dehydrogenase; *TGF-β1–3*, transforming growth factor beta 1–3; *IL-1β*, interleukin-1 beta; *IL-15*, interleukin-15; *IHNBA*, inhibin beta A chain; *VEGFA*, vascular endothelial growth factor A; *SLURP1*, secreted Ly-6_uPAR-related protein 1.

**Table 3:** Changes in gene expression in tears of patients who underwent PRK, FS-LASIK, and SMILE over 180 days of observation.

mRNA	PRK				FS-LASIK				SMILE			
	1 Day	7 Days	30 Days	180 Days	1 Day	7 Days	30 Days	180 Days	1 Day	7 Days	30 Days	180 Days
*TGF-β1*	2.42 ± 0.19 ^A,B,C^	1.76 ± 0.23 ^D,E^	1.27 ± 0.21	1.18 ± 0.44	3.45 ± 0.31	3.12 ± 0.23 ^E^	2.98 ± 0.11 ^F^	1.19 ± 0.12	1.27 ± 0.27	1.14 ± 0.12	1.21 ± 0.31	1.27 ± 0.17
*TGF-β2*	3.45 ± 0.21 ^A,B,C^	2.14 ± 0.31 ^D,E^	1.09 ± 0.23 ^F^	1.45 ± 0.31	4.98 ± 0.35	4.56 ± 0.25 ^E^	4.12 ± 0.78 ^F^	1.12 ± 0.11	1.13 ± 0.16	1.02 ± 0.17 ^E^	1.05 ± 0.18	1.12 ± 032
*TGF-β3*	1.13 ± 0.12 ^B,C^	1.03 ± 0.19 ^D,E^	0.88 ± 0.13 ^F^	0.78 ± 0.13	2.01 ± 0.19	2.13 ± 0.43 ^E^	2.34 ± 0.24 ^F^	1.09 ± 0.21	0.76 ± 0.12	0.65 ± 0.08	0.67 ± 0.17	0.87 ± 0.23
*IL-1β*	3.14 ± 0.19 ^B,C^	3.02 ± 0.76 ^D,E^	2.54 ± 0.24	1.98 ± 0.23	1.98 ± 0.24 ^A,B,C^	1.11 ± 0.18	0.97 ± 0.43	0.96 ± 0.37	1.04 ± 0.31	1.12 ± 0.25	1.13 ± 0.13	1.09 ± 0.07
*IL-15*	4.65 ± 0.65	4.98 ± 0.46 ^E^	4.54 ± 0.76 ^F^	3.21 ± 0.41	3.55 ± 0.37 ^A,B,C^	4.99 ± 0.28 ^D,E^	1.98 ± 0.19	2.13 ± 0.24	2.09 ± 0.23	1.45 ± 0.13 ^D^	1.12 ± 0.08	1.32 ± 0.19
*INHBA*	0.65 ± 0.12 ^A,B,C^	1.09 ± 0.19	1.56 ± 0.21 ^F^	1.13 ± 0.34	0.56 ± 0.08 ^A,B,C^	1.01 ± 0.17	1.22 ± 0.12	1.23 ± 0.44	1.09 ± 0.08	1.15 ± 0.17	1.01 ± 0.04	1.01 ± 0.12
*VEGFA*	2.98 ± 0.23	2.17 ± 0.23 ^D,E^	3.01 ± 0.26 ^F^	1.16 ± 0.46	3.45 ± 1.09 ^C^	3.46 ± 0.41 ^D,E^	2.09 ± 0.87 ^F^	1.54 ± 0.32	1.87 ± 0.31	1.76 ± 0.23	1.31 ± 0.16	1.45 ± 0.44
*SLURP1*	1.04 ± 0.41	1.07 ± 0.11	1.17 ± 0.12	1.02 ± 0.12	1.54 ± 0.32	1.21 ± 0.19	1.18 ± 0.17	1.09 ± 0.10	1.08 ± 0.29	1.15 ± 0.21	1.21 ± 0.21	1.03 ± 0.09

*TGF-β1–3*, transforming growth factor beta 1-3; *IL-1B*, interleukin-1 beta; *IL-15*, interleukin-15; *IHNBA*, inhibin beta A chain; *VEGFA*, vascular endothelial growth factor A; *SLURP1*, secreted Ly-6_uPAR-related protein 1; PRK, photorefractive keratectomy; FS-LASIK, femtosecond-assisted laser in situ keratomileusis; SMILE, refractive lenticule extraction with small incision. Statistically significant differences (*p* < 0.05) between days 1 and 7 (A), between days 1 and 30 (B), between days 1 and 180 (C), between days 7 and 30 (D), between days 7 and 180 (E), and between days 30 and 180 (F). Data are presented as means ± standard deviations.

## Data Availability

The original contributions presented in the study are included in the article/Supplementary Materials, and further inquiries can be directed to the corresponding author.

## Supplementary Materials

The following supporting information can be downloaded at https://www.mdpi.com/article/10.3390/biomedicines12102289/s1, Table S1: The raw results of the protein-level concentrations of the assessed cytokines in each patient undergoing PRK, FS-LASIK, and SMILE, Table S2: The detailed results of the post hoc test of the individual comparisons of the concentrations of a given cytokine at follow-up depending on the LVC used, Table S3: Comparison of IL-1β, IL-15, and SLURP1 concentrations in patients before and after PRK, FS-LASIK, and SMILE based on participants’ gender.
