# Supplementary material for: Comparative Analysis of Corneal Wound Healing: Differential Molecular Responses in Tears Following PRK, FS-LASIK, and SMILE Procedures
Source: Biomedicines. 2024 Oct 9;12(10):2289. doi: 10.3390/biomedicines12102289 (PMC11505177; doi:10.3390/biomedicines12102289)
Supplement: Supplementary file 1 [file biomedicines-12-02289-s001.zip › Table S1.pdf]

Table S1: The raw results of the protein-level concentrations of the assessed cytokines in each patient undergoing PRK, FS-LASIK, and SMILE

Procedure	Cytokine [pg/mL]	0 day	1 day	7 days	30 days	180 days
PRK	TGF-B1	45.26	69.94	67.25	47.70	36.06
	TGF-B1	42.33	82.11	66.66	51.69	40.53
	TGF-B1	35.43	76.96	69.87	49.34	42.16
	TGF-B1	40.49	82.26	63.82	49.02	38.75
	TGF-B1	46.38	67.98	76.32	50.21	51.12
	TGF-B1	40.34	67.72	52.98	44.91	38.51
	TGF-B1	44.26	84.97	58.42	61.93	36.48
	TGF-B1	37.12	78.17	69.33	54.63	39.93
	TGF-B1	39.89	82.94	60.63	52.82	51.44
	TGF-B1	46.38	74.99	60.58	53.48	36.54
	TGF-B1	46.09	80.59	71.21	47.72	45.12
	TGF-B1	49.04	79.18	69.89	55.31	45.14
	TGF-B1	48.67	65.94	66.33	47.30	42.10
	TGF-B1	45.01	79.85	74.42	64.73	46.12
	TGF-B1	48.82	78.24	62.99	55.76	37.10
	TGF-B1	36.29	74.86	67.12	46.59	39.72
	TGF-B1	45.25	73.73	78.42	43.57	44.81
	TGF-B1	32.77	74.19	62.73	49.90	41.20
	TGF-B1	47.55	73.29	61.19	62.23	38.27
	TGF-B1	31.97	78.23	67.28	62.72	33.23
PRK		0 day	1 day	7 days	30 days	180 days
	TGFB2	42.63	85.55	69.78	55.06	53.61
	TGFB2	32.97	93.63	65.99	64.14	49.37
	TGFB2	39.69	86.93	73.95	66.71	48.82
	TGFB2	47.60	88.93	67.40	58.49	50.62
	TGFB2	47.25	95.10	67.48	58.05	47.62
	TGFB2	44.12	86.23	65.94	59.04	54.48
	TGFB2	50.45	89.77	68.88	50.98	47.79
	TGFB2	54.90	86.54	68.14	62.66	50.50
	TGFB2	50.21	87.20	64.68	55.77	47.87
	TGFB2	44.46	83.96	65.21	58.17	46.95
	TGFB2	43.60	77.72	60.23	55.69	43.14
	TGFB2	48.06	83.38	63.70	65.08	36.81
	TGFB2	41.94	82.02	71.71	57.69	44.79
	TGFB2	43.03	83.50	77.05	64.62	47.61
	TGFB2	44.29	85.04	58.41	60.39	38.91
	TGFB2	47.07	89.16	66.62	57.71	49.76
	TGFB2	47.31	89.41	63.99	44.53	47.21
	TGFB2	46.13	83.55	65.20	60.53	51.17
	TGFB2	52.22	85.34	58.27	59.54	45.77
	TGFB2	54.39	86.50	69.91	66.63	44.10

		0 day	1 day	7 days	30 days	180 days
PRK	TGFB3	41.20	61.40	82.24	48.62	43.82
	TGFB3	39.58	58.56	81.54	60.88	49.95
	TGFB3	41.14	48.69	84.07	50.49	45.42
	TGFB3	38.54	54.25	78.54	41.87	46.23
	TGFB3	51.31	64.63	79.88	46.90	42.45
	TGFB3	31.76	59.48	76.50	55.20	46.54
	TGFB3	43.05	62.68	79.31	56.27	52.88
	TGFB3	32.30	64.64	77.67	52.35	52.43
	TGFB3	46.05	61.21	75.70	53.75	37.82
	TGFB3	45.12	52.52	80.92	45.68	42.35
	TGFB3	38.28	63.19	84.95	48.88	40.56
	TGFB3	44.14	54.84	72.64	45.53	41.50
	TGFB3	44.06	54.65	81.09	53.48	46.84
	TGFB3	35.15	46.76	82.56	54.63	45.53
	TGFB3	41.12	58.98	74.21	53.96	45.50
	TGFB3	39.94	57.29	76.69	60.11	46.58
	TGFB3	43.22	59.61	78.18	60.29	39.65
	TGFB3	33.01	59.54	70.29	53.66	42.09
	TGFB3	51.43	45.73	70.35	49.40	50.52
	TGFB3	46.33	52.78	66.61	43.00	45.62

		0 day	1 day	7 days	30 days	180 days
PRK	IL1B	58.64	68.33	77.84	43.64	57.17
	IL1B	69.01	72.73	81.67	38.65	58.13
	IL1B	62.79	62.73	80.33	36.16	55.63
	IL1B	63.56	73.94	73.16	47.19	52.17
	IL1B	67.23	74.62	80.22	33.05	51.37
	IL1B	66.95	70.59	78.91	37.40	57.00
	IL1B	68.58	63.17	66.75	31.89	52.06
	IL1B	64.31	71.05	70.56	37.48	51.55
	IL1B	59.54	72.02	80.14	41.52	51.15
	IL1B	62.15	73.35	78.51	43.38	49.23
	IL1B	56.49	74.03	75.17	43.28	54.75
	IL1B	68.70	66.85	72.27	42.20	48.25
	IL1B	69.11	77.51	83.34	34.91	55.15
	IL1B	65.46	61.70	73.34	36.81	45.28
	IL1B	68.01	70.05	66.54	36.34	58.75
	IL1B	72.22	76.54	71.86	40.88	59.82
	IL1B	67.41	67.31	76.75	33.66	54.04
	IL1B	59.45	69.95	84.06	34.82	59.88
	IL1B	61.15	69.75	75.91	38.78	55.42
	IL1B	66.30	74.35	78.46	53.90	55.65

		0 day	1 day	7 days	30 days	180 days
	IL15	10.19	27.09	12.43	7.34	18.59
	IL15	8.16	16.54	14.71	12.93	13.23
	IL15	18.08	20.87	18.78	16.82	10.72
	IL15	5.49	21.32	12.02	17.47	15.45
	IL15	14.87	23.41	22.69	13.09	16.46
	IL15	2.31	18.74	23.68	23.22	6.69
	IL15	14.06	16.95	19.32	10.50	9.69
	IL15	9.24	13.51	13.63	6.78	10.57
	IL15	20.06	23.82	11.49	4.60	4.03
**PRK**	IL15	15.50	27.14	21.10	10.81	12.63
	IL15	17.37	18.55	8.20	14.60	8.14
	IL15	3.45	17.08	25.22	16.88	6.10
	IL15	10.33	22.11	19.20	-1.35	9.70
	IL15	11.20	17.80	10.96	18.45	15.78
	IL15	11.54	22.56	15.91	24.24	10.79
	IL15	4.94	27.13	20.88	16.76	11.26
	IL15	13.50	19.08	14.42	17.26	8.14
	IL15	9.37	16.08	8.55	9.55	17.63
	IL15	8.24	11.34	23.31	14.51	7.77
	IL15	15.29	27.01	21.53	8.23	16.93
		0 day	1 day	7 days	30 days	180 days
	INHBA	19.83	13.73	12.01	12.08	11.85
	INHBA	9.93	13.56	15.75	-0.37	16.49
	INHBA	12.96	12.51	18.58	3.07	13.13
	INHBA	19.52	5.22	9.58	14.74	15.70
	INHBA	7.01	12.79	9.21	10.18	5.04
	INHBA	10.52	9.08	20.35	7.26	6.61
	INHBA	12.17	-2.52	4.62	11.58	11.43
	INHBA	19.51	15.34	4.96	6.62	7.32
	INHBA	11.61	20.72	6.14	9.93	11.60
**PRK**	INHBA	7.93	6.33	12.68	5.02	12.14
	INHBA	10.27	17.08	13.29	15.64	13.93
	INHBA	12.50	5.87	16.89	8.88	8.81
	INHBA	7.06	13.28	2.58	13.64	2.92
	INHBA	14.31	14.60	16.14	11.81	4.65
	INHBA	4.20	10.23	20.96	16.28	9.22
	INHBA	8.45	18.97	23.45	8.70	12.10
	INHBA	5.64	-0.09	14.48	14.98	6.96
	INHBA	6.48	4.33	8.28	5.94	5.38
	INHBA	11.91	9.50	9.92	9.99	8.97
	INHBA	13.92	0.48	9.43	8.83	6.20

		0 day	1 day	7 days	30 days	180 days
PRK	VEGFA	66.01	111.05	116.91	101.03	69.66
	VEGFA	68.59	109.47	102.92	104.12	63.47
	VEGFA	66.38	106.84	102.26	101.19	66.95
	VEGFA	58.61	101.48	109.07	99.81	64.50
	VEGFA	72.20	102.25	115.19	92.66	57.53
	VEGFA	69.55	114.93	97.37	100.34	62.80
	VEGFA	69.00	112.07	100.56	106.37	71.80
	VEGFA	65.34	108.46	104.91	93.58	61.12
	VEGFA	66.53	109.67	104.62	95.90	66.05
	VEGFA	66.82	110.40	112.66	106.23	69.05
	VEGFA	71.87	113.68	109.30	94.30	73.18
	VEGFA	70.52	103.31	100.47	83.86	68.30
	VEGFA	75.75	102.61	110.30	106.81	71.07
	VEGFA	61.70	109.47	110.34	96.12	73.34
	VEGFA	70.44	105.40	111.88	103.52	67.95
	VEGFA	65.30	111.45	115.89	97.52	70.52
	VEGFA	75.83	103.88	116.15	97.59	70.45
	VEGFA	76.20	111.14	104.33	87.37	67.37
	VEGFA	71.53	119.01	107.37	104.10	69.89
	VEGFA	72.88	113.69	100.56	100.85	67.62

		0 day	1 day	7 days	30 days	180 days
PRK	SLURP1	29.09	27.95	26.83	38.18	31.31
	SLURP1	37.82	34.21	26.19	29.77	36.86
	SLURP1	40.54	32.05	26.70	32.60	38.80
	SLURP1	37.34	28.50	32.80	35.76	34.50
	SLURP1	36.92	35.24	28.71	33.97	44.81
	SLURP1	34.34	30.86	23.49	22.58	27.69
	SLURP1	26.07	33.69	38.30	33.73	31.13
	SLURP1	37.46	39.71	35.53	38.12	38.06
	SLURP1	37.22	35.09	37.17	35.16	36.06
	SLURP1	31.25	32.66	39.75	26.99	39.66
	SLURP1	35.36	35.82	30.60	38.07	39.05
	SLURP1	40.23	33.09	38.31	35.70	32.84
	SLURP1	33.95	20.96	44.12	34.74	34.23
	SLURP1	37.45	22.32	39.83	42.48	31.42
	SLURP1	26.76	31.52	25.69	40.31	31.59
	SLURP1	29.96	39.23	22.35	30.95	35.35
	SLURP1	33.45	25.79	26.45	30.51	38.17
	SLURP1	42.35	30.08	27.09	33.31	34.16
	SLURP1	30.39	31.44	36.31	34.43	42.35
	SLURP1	39.14	28.77	32.18	30.09	34.64

	Cytokine	0 day	1 day	7 days	30 days	180 days
FS-LASIK	TGF-B1	35.27	86.20	83.00	71.29	45.38
	TGF-B1	33.84	88.37	93.76	63.72	51.44
	TGF-B1	48.48	85.94	85.54	64.00	51.23
	TGF-B1	40.46	94.68	85.37	64.84	48.03
	TGF-B1	40.10	89.59	83.23	68.01	36.34
	TGF-B1	39.35	88.80	80.32	61.53	42.44
	TGF-B1	36.43	96.69	89.25	66.76	51.15
	TGF-B1	47.02	98.05	87.35	72.29	53.13
	TGF-B1	40.18	92.90	85.96	67.48	41.27
	TGF-B1	29.50	94.16	85.78	68.76	43.43
	TGF-B1	35.70	93.47	85.81	72.49	51.98
	TGF-B1	45.67	87.79	86.92	61.59	58.82
	TGF-B1	44.58	90.33	83.78	71.78	51.39
	TGF-B1	41.04	91.37	87.59	63.78	49.62
	TGF-B1	34.60	97.45	97.52	63.76	51.03
	TGF-B1	38.08	87.97	92.36	63.36	50.71
	TGF-B1	40.52	95.18	95.09	65.68	46.87
	TGF-B1	42.48	92.16	87.04	72.01	53.03
	TGF-B1	50.97	95.18	86.63	67.47	42.71
	TGF-B1	45.16	96.57	85.01	72.53	41.18
	TGF-B1	43.76	98.85	86.91	70.30	49.56
	TGF-B1	41.01	99.22	91.67	59.83	49.09
	TGF-B1	30.05	88.24	84.44	62.34	51.30
	TGF-B1	49.11	95.72	80.24	59.29	41.89
	TGF-B1	38.93	100.55	90.55	70.66	41.34
	TGF-B1	46.41	94.53	88.48	74.30	53.52
	TGF-B1	41.14	86.96	84.37	74.11	47.97
	TGF-B1	35.85	100.50	83.57	66.26	54.75
	TGF-B1	43.73	84.86	90.38	78.12	39.68
	TGF-B1	43.07	92.24	89.76	69.86	54.49
	TGF-B1	39.51	96.82	81.77	73.09	49.83
	TGF-B1	41.39	91.92	88.20	71.48	49.59
	TGF-B1	42.81	89.48	88.67	73.70	43.00
	TGF-B1	38.93	93.11	91.16	71.84	47.91
	TGF-B1	39.62	89.16	78.94	68.13	50.22
	TGF-B1	43.10	95.37	74.10	66.73	42.44
	TGF-B1	41.17	83.35	81.55	71.33	39.70
	TGF-B1	41.42	102.46	93.70	61.64	48.28

	TGF-B1	56.60	96.57	86.90	68.00	50.31
	TGF-B1	43.20	96.38	80.09	66.89	47.65
	TGF-B1	42.60	94.03	81.42	61.19	46.27
	TGF-B1	36.61	101.22	83.21	69.52	54.69
	TGF-B1	40.42	90.35	77.74	62.28	46.89
	TGF-B1	38.26	99.11	84.50	70.95	45.14
	TGF-B1	38.15	79.66	84.99	67.22	51.67
	TGF-B1	42.17	94.63	79.56	66.22	45.81
	TGF-B1	45.38	84.14	90.89	68.42	52.25
	TGF-B1	42.73	95.71	85.23	67.07	58.66
	TGF-B1	35.76	91.23	83.66	75.85	53.91
	TGF-B1	41.07	93.64	88.62	66.10	50.44
	Cytokine	0 day	1 day	7 days	30 days	180 days
	TGFB2	57.41	111.12	101.90	94.16	64.40
	TGFB2	52.85	112.20	98.60	89.35	62.05
	TGFB2	59.28	108.69	100.67	77.78	69.84
	TGFB2	56.93	110.08	93.02	93.68	51.82
	TGFB2	56.56	115.60	111.14	91.50	59.81
	TGFB2	52.07	102.91	100.89	86.56	61.79
	TGFB2	47.91	99.38	99.26	81.84	68.95
	TGFB2	50.73	117.89	100.80	77.48	50.90
	TGFB2	51.07	116.11	94.87	85.30	60.72
	TGFB2	50.83	104.78	91.72	82.68	56.03
	TGFB2	58.05	118.80	96.94	84.30	53.88
	TGFB2	52.00	115.73	105.99	89.44	65.28
	TGFB2	66.07	113.42	93.64	81.15	67.04
**FS-LASIK**	TGFB2	50.31	101.92	92.29	88.13	60.65
	TGFB2	58.41	101.78	96.66	90.27	67.94
	TGFB2	59.19	108.47	96.11	94.18	56.32
	TGFB2	48.86	115.91	103.04	92.57	62.15
	TGFB2	55.57	103.61	106.81	91.12	68.07
	TGFB2	45.29	108.62	99.67	86.75	65.60
	TGFB2	49.30	113.59	108.51	83.15	54.80
	TGFB2	49.75	108.30	96.42	90.37	67.66
	TGFB2	52.04	114.48	96.76	86.46	59.78
	TGFB2	52.07	105.68	104.10	89.07	65.15
	TGFB2	55.21	114.29	97.08	87.57	63.17
	TGFB2	44.50	103.64	99.85	87.02	55.93
	TGFB2	55.54	109.70	99.76	88.46	54.07
	TGFB2	56.49	115.97	101.63	88.41	57.51
	TGFB2	55.92	114.66	93.14	88.48	53.01
	TGFB2	56.51	101.91	108.62	78.76	60.18

	TGFB2	61.14	103.83	103.27	93.12	60.82
	TGFB2	54.62	112.55	106.51	91.46	66.73
	TGFB2	58.63	101.49	102.93	86.42	66.56
	TGFB2	59.82	113.77	86.52	81.68	57.92
	TGFB2	62.17	104.86	102.88	89.69	69.51
	TGFB2	55.06	120.24	102.55	82.58	55.05
	TGFB2	54.84	107.27	108.79	80.61	65.16
	TGFB2	58.81	112.82	104.35	83.30	58.10
	TGFB2	57.48	102.21	107.65	92.24	57.63
	TGFB2	52.62	113.92	97.28	89.93	63.40
	TGFB2	56.52	109.17	109.72	83.83	63.96
	TGFB2	56.95	108.61	97.49	85.85	69.78
	TGFB2	61.07	109.55	96.20	82.70	65.34
	TGFB2	47.36	106.86	108.10	91.11	64.94
	TGFB2	50.94	106.50	94.19	88.69	61.47
	TGFB2	60.00	105.70	99.94	102.84	61.92
	TGFB2	54.28	119.11	106.86	83.02	67.72
	TGFB2	48.68	107.59	93.36	84.44	66.11
	TGFB2	61.48	104.91	96.70	91.95	64.97
	TGFB2	59.88	106.01	109.38	98.63	59.54
	TGFB2	57.54	113.82	101.04	93.13	56.27
	Cytokine	0 day	1 day	7 days	30 days	180 days
**FS-LASIK**	TGFB3	53.10	100.74	71.23	54.05	47.91
	TGFB3	44.84	96.65	64.15	56.58	46.73
	TGFB3	47.27	102.41	52.99	58.23	45.13
	TGFB3	52.27	98.12	74.73	55.86	46.88
	TGFB3	44.83	101.27	66.51	55.61	57.50
	TGFB3	40.74	102.20	65.00	53.26	48.21
	TGFB3	39.28	96.79	68.96	46.94	44.74
	TGFB3	44.59	100.25	58.23	51.03	44.76
	TGFB3	51.11	89.10	66.66	52.31	53.08
	TGFB3	50.06	99.21	62.81	51.04	61.18
	TGFB3	49.65	99.60	73.60	54.34	51.11
	TGFB3	47.50	101.29	62.31	59.78	53.29
	TGFB3	39.60	106.81	71.32	53.68	52.48
	TGFB3	48.21	93.19	66.76	66.13	53.06
	TGFB3	48.84	100.81	68.39	59.07	51.87
	TGFB3	45.00	92.44	62.28	52.54	51.81
	TGFB3	50.61	94.96	61.09	61.95	49.35
	TGFB3	51.77	102.38	54.99	55.52	56.52
	TGFB3	45.71	93.70	68.49	50.07	59.62
	TGFB3	45.25	103.91	70.46	66.39	50.01

	TGFB3	45.72	98.13	72.02	58.60	51.50
	TGFB3	43.00	98.18	61.90	62.03	42.34
	TGFB3	46.22	96.57	65.03	56.46	32.32
	TGFB3	39.74	103.72	60.00	54.86	43.54
	TGFB3	40.74	104.95	64.59	53.41	44.14
	TGFB3	44.86	99.27	64.58	56.43	55.35
	TGFB3	56.60	92.03	68.13	57.35	43.22
	TGFB3	44.09	104.91	66.21	53.12	55.40
	TGFB3	46.54	102.24	70.40	50.88	54.05
	TGFB3	52.63	90.85	61.63	54.31	56.43
	TGFB3	51.66	97.96	67.45	53.32	41.37
	TGFB3	50.28	104.70	65.11	54.62	49.24
	TGFB3	48.26	101.02	66.28	54.11	53.64
	TGFB3	46.95	91.34	67.43	56.09	59.64
	TGFB3	48.87	86.59	74.29	69.03	42.97
	TGFB3	46.63	106.08	66.85	70.06	49.32
	TGFB3	40.00	101.51	70.11	54.59	37.78
	TGFB3	52.16	98.54	63.41	59.52	51.30
	TGFB3	53.75	88.20	64.49	54.37	51.25
	TGFB3	43.09	94.80	60.96	44.25	61.06
	TGFB3	49.24	88.71	67.72	60.08	51.34
	TGFB3	40.77	91.51	67.01	54.60	50.35
	TGFB3	59.90	100.82	63.24	55.31	48.98
	TGFB3	48.55	96.71	71.11	51.24	55.70
	TGFB3	43.81	106.77	58.38	56.84	46.16
	TGFB3	45.96	95.11	62.46	46.62	41.41
	TGFB3	52.44	95.22	58.34	41.69	46.13
	TGFB3	43.41	105.24	60.59	54.02	43.15
	TGFB3	47.79	92.67	72.31	53.83	49.42
	TGFB3	61.76	107.59	74.61	57.58	54.75
**FS-LASIK**	Cytokine	0 day	1 day	7 days	30 days	180 days
	IL1B	54.66	63.08	51.10	62.25	55.14
	IL1B	56.00	56.86	50.71	52.89	53.72
	IL1B	62.03	62.29	51.32	54.71	59.84
	IL1B	56.13	67.14	58.79	48.57	50.23
	IL1B	54.81	61.96	62.16	51.96	52.67
	IL1B	62.48	58.48	63.19	61.29	60.87
	IL1B	56.06	67.49	56.32	56.46	69.58
	IL1B	64.35	53.73	45.26	57.30	53.08
	IL1B	68.59	67.70	50.70	58.21	53.78
	IL1B	61.23	55.43	55.52	57.50	52.04
	IL1B	65.17	64.87	56.02	60.25	57.29

	IL1B	59.69	56.77	56.16	52.09	59.01
	IL1B	54.42	53.58	63.99	52.24	57.68
	IL1B	67.91	62.21	61.28	59.22	58.26
	IL1B	59.34	54.20	59.81	53.17	64.06
	IL1B	61.03	54.11	66.83	58.48	56.33
	IL1B	55.37	48.94	56.74	56.40	62.85
	IL1B	69.84	62.57	60.13	56.26	57.26
	IL1B	67.20	53.18	55.84	52.16	53.90
	IL1B	52.09	53.24	51.65	52.33	49.04
	IL1B	54.72	47.62	48.24	53.62	62.19
	IL1B	60.59	52.49	56.84	64.94	57.64
	IL1B	53.95	58.71	61.84	57.28	64.34
	IL1B	62.21	59.81	60.44	51.68	58.56
	IL1B	64.72	57.48	56.34	59.44	52.79
	IL1B	59.23	54.25	46.85	49.68	55.14
	IL1B	59.05	66.01	64.08	54.58	61.28
	IL1B	66.40	61.85	56.52	55.98	51.88
	IL1B	56.97	59.48	58.94	53.17	57.03
	IL1B	59.63	53.25	57.70	59.73	63.13
	IL1B	60.67	58.26	54.87	58.53	64.06
	IL1B	63.00	55.78	57.70	52.18	64.79
	IL1B	63.65	61.81	65.64	59.16	59.47
	IL1B	66.70	68.66	52.11	62.91	62.75
	IL1B	57.06	57.97	53.37	47.29	42.05
	IL1B	62.28	61.69	60.95	46.18	51.31
	IL1B	58.55	59.65	53.23	66.46	60.76
	IL1B	54.70	62.47	56.65	58.73	53.28
	IL1B	59.84	59.55	53.56	62.31	66.72
	IL1B	59.99	60.95	65.03	64.08	64.41
	IL1B	61.03	60.38	53.36	53.76	54.50
	IL1B	56.63	53.44	56.22	56.09	54.85
	IL1B	50.25	61.07	60.95	54.35	54.64
	IL1B	65.44	46.63	52.11	64.25	64.60
	IL1B	52.41	61.84	56.37	51.20	56.59
	IL1B	65.32	55.17	65.49	46.32	65.79
	IL1B	62.44	54.78	48.29	49.69	43.97
	IL1B	63.50	58.59	51.58	49.93	63.80
	IL1B	69.16	61.56	55.93	58.54	49.88
	IL1B	63.74	63.80	52.58	58.20	64.31
**FS-LASIK**	Cytokine	0 day	1 day	7 days	30 days	180 days
	IL15	12.17	27.21	53.12	28.23	26.59
	IL15	8.64	32.13	50.38	36.77	23.93

	IL15	15.44	21.13	56.44	38.62	30.12
	IL15	9.19	24.85	48.27	17.68	32.86
	IL15	12.40	28.24	56.78	32.53	25.12
	IL15	11.57	17.54	49.62	23.95	22.08
	IL15	9.78	29.74	47.32	27.75	23.18
	IL15	19.05	33.52	40.86	34.90	25.41
	IL15	7.87	27.65	48.03	36.53	31.86
	IL15	15.03	32.77	42.64	34.76	25.06
	IL15	4.15	22.65	47.18	25.92	28.25
	IL15	15.09	34.40	58.39	31.11	32.39
	IL15	21.18	19.03	47.82	34.17	21.69
	IL15	5.46	30.67	45.43	25.50	20.61
	IL15	14.18	27.17	42.09	28.45	24.21
	IL15	15.96	28.86	43.89	26.97	28.91
	IL15	6.09	30.00	41.61	28.57	32.96
	IL15	19.93	35.49	53.82	29.51	30.04
	IL15	3.25	26.59	55.68	31.72	30.09
	IL15	9.03	17.41	45.02	28.55	15.34
	IL15	12.65	30.58	38.46	34.85	21.03
	IL15	16.93	28.31	63.15	36.59	19.24
	IL15	9.77	21.57	60.81	23.82	30.46
	IL15	24.31	30.61	47.97	30.48	19.89
	IL15	6.66	23.02	53.87	28.64	32.23
	IL15	10.61	27.24	52.33	32.02	25.36
	IL15	18.99	20.15	46.61	25.82	21.44
	IL15	10.74	32.43	44.95	29.00	16.92
	IL15	11.66	30.81	38.13	27.96	27.48
	IL15	15.49	21.62	50.79	36.52	22.08
	IL15	6.68	32.54	47.75	27.06	23.58
	IL15	16.17	23.59	44.47	37.74	26.26
	IL15	6.35	20.56	49.35	25.49	25.24
	IL15	5.88	34.71	48.22	22.26	23.98
	IL15	11.69	29.70	50.38	33.10	25.16
	IL15	8.78	26.50	54.05	35.20	27.21
	IL15	19.08	29.09	49.14	36.28	30.17
	IL15	15.02	28.17	45.87	29.72	25.91
	IL15	8.81	23.71	46.66	37.32	25.44
	IL15	12.98	26.36	44.81	39.78	34.52
	IL15	5.71	37.55	50.90	34.59	23.48
	IL15	5.87	26.30	40.43	24.91	28.27
	IL15	16.53	28.34	41.96	32.05	21.10
	IL15	14.38	23.34	46.96	24.69	28.93

	IL15	11.58	27.58	50.04	28.54	24.22
	IL15	3.34	20.26	54.43	29.69	32.92
	IL15	6.59	25.44	34.53	29.60	16.81
	IL15	13.86	18.61	50.87	20.98	31.16
	IL15	0.72	30.89	49.28	31.95	19.10
	IL15	13.07	25.89	52.98	32.97	24.81
	Cytokine	0 day	1 day	7 days	30 days	180 days
**FS-LASIK**	IHNB	19.01	71.51	62.85	29.72	16.04
	IHNB	7.04	73.42	61.86	25.57	26.60
	IHNB	17.21	71.83	70.32	17.54	16.33
	IHNB	13.69	79.30	56.99	17.56	18.37
	IHNB	16.61	68.40	57.48	22.73	14.06
	IHNB	9.98	71.61	65.01	15.77	8.30
	IHNB	13.05	71.89	55.07	31.05	20.58
	IHNB	22.08	73.10	47.73	31.41	20.95
	IHNB	17.19	75.13	70.98	26.00	19.60
	IHNB	16.16	79.54	54.50	27.21	15.97
	IHNB	14.18	74.02	65.44	27.64	26.10
	IHNB	16.35	81.56	65.58	29.88	13.51
	IHNB	22.79	83.69	57.47	32.88	18.90
	IHNB	14.40	82.99	61.86	25.91	14.54
	IHNB	14.94	70.13	59.26	33.63	19.01
	IHNB	11.60	71.87	54.06	20.80	12.39
	IHNB	8.25	87.67	61.59	25.54	19.27
	IHNB	18.59	72.92	65.44	17.33	22.23
	IHNB	10.32	79.17	69.44	27.85	24.44
	IHNB	2.16	72.76	62.16	17.89	10.94
	IHNB	18.26	76.95	64.17	25.99	21.16
	IHNB	13.95	80.52	60.84	23.59	24.75
	IHNB	12.61	75.29	65.09	23.19	17.43
	IHNB	10.32	73.64	68.25	9.87	23.93
	IHNB	10.16	71.54	68.27	20.73	14.58
	IHNB	11.40	82.31	64.37	15.02	9.59
	IHNB	11.85	72.64	62.87	20.25	25.28
	IHNB	11.76	78.01	65.42	27.74	19.87
	IHNB	16.26	79.38	47.90	31.86	11.87
	IHNB	2.80	76.00	59.91	22.20	25.87
	IHNB	13.77	68.82	60.94	21.41	19.67
	IHNB	20.27	79.88	65.47	26.45	24.01
	IHNB	13.88	79.29	69.49	25.14	23.00
	IHNB	19.96	76.39	66.53	22.43	26.76
	IHNB	6.60	79.33	56.19	24.38	14.21

	IHNB	15.79	79.70	64.94	21.40	24.22
	IHNB	10.04	88.50	57.46	24.66	26.02
	IHNB	13.68	71.70	62.73	35.47	16.07
	IHNB	20.56	74.78	70.58	21.12	18.34
	IHNB	13.67	71.83	68.63	30.82	22.48
	IHNB	8.69	73.53	55.72	18.67	16.83
	IHNB	8.93	73.41	61.46	32.28	14.80
	IHNB	13.10	80.73	59.33	23.23	24.30
	IHNB	12.64	74.01	58.62	21.26	13.55
	IHNB	11.79	92.19	69.30	29.80	18.67
	IHNB	21.32	67.05	70.05	32.88	20.42
	IHNB	26.82	70.50	55.41	24.23	25.18
	IHNB	8.84	79.78	57.23	26.47	19.16
	IHNB	10.07	74.62	65.99	25.26	20.59
	IHNB	11.76	67.15	59.30	26.34	15.73
**FS-LASIK**	Cytokine	0 day	1 day	7 days	30 days	180 days
	VEGFA	54.93	59.47	54.00	49.95	51.54
	VEGFA	58.63	57.53	61.45	47.91	51.61
	VEGFA	58.37	62.65	62.56	49.20	51.52
	VEGFA	56.62	63.82	67.47	51.64	52.45
	VEGFA	49.46	58.84	67.35	61.06	56.40
	VEGFA	65.39	60.48	60.66	56.23	46.88
	VEGFA	63.85	61.64	56.95	47.56	51.61
	VEGFA	59.86	56.58	54.53	59.63	51.67
	VEGFA	52.10	62.76	51.76	50.86	48.77
	VEGFA	58.29	59.25	61.27	50.48	45.30
	VEGFA	59.17	55.22	63.11	51.55	52.91
	VEGFA	63.05	63.08	69.44	60.04	51.64
	VEGFA	59.69	59.13	57.70	54.89	50.04
	VEGFA	58.64	60.43	55.12	56.89	59.10
	VEGFA	56.60	61.47	58.63	57.29	59.76
	VEGFA	57.37	64.32	63.56	60.78	56.56
	VEGFA	55.27	54.69	53.84	52.86	53.91
	VEGFA	64.43	56.77	52.40	52.16	50.21
	VEGFA	56.01	52.91	63.57	56.71	54.07
	VEGFA	46.17	56.73	58.41	50.09	48.29
	VEGFA	55.02	68.83	60.19	58.57	55.17
	VEGFA	55.01	61.63	64.00	59.63	49.71
	VEGFA	57.50	67.83	60.85	58.41	54.86
	VEGFA	53.62	65.68	59.14	59.54	47.10
	VEGFA	59.05	68.28	55.13	53.89	48.82
	VEGFA	58.35	68.80	59.07	63.33	53.86

	VEGFA	58.31	59.89	53.54	53.93	56.99
	VEGFA	59.24	53.98	57.25	56.22	56.50
	VEGFA	51.17	60.31	55.83	55.17	52.51
	VEGFA	52.71	66.39	56.64	54.76	50.48
	VEGFA	48.87	66.74	56.72	49.13	57.08
	VEGFA	55.82	55.59	58.68	57.52	58.75
	VEGFA	47.77	56.03	64.84	51.59	57.22
	VEGFA	51.42	65.37	59.32	48.75	49.52
	VEGFA	55.17	70.12	55.36	57.36	59.05
	VEGFA	56.41	71.16	52.92	51.50	55.56
	VEGFA	62.88	66.71	70.46	59.14	58.95
	VEGFA	56.29	55.27	52.74	62.08	45.76
	VEGFA	54.09	57.06	51.87	56.29	50.31
	VEGFA	50.75	56.32	52.40	59.18	47.57
	VEGFA	63.05	59.13	57.49	57.54	50.96
	VEGFA	67.16	63.70	56.57	63.28	55.66
	VEGFA	57.24	54.50	53.25	48.69	58.47
	VEGFA	46.45	58.30	63.40	61.16	58.16
	VEGFA	61.21	63.90	58.81	60.56	56.30
	VEGFA	61.39	67.18	57.64	42.55	46.30
	VEGFA	52.16	53.08	63.49	50.43	54.32
	VEGFA	63.06	53.20	63.44	48.15	43.44
	VEGFA	63.51	56.50	62.78	60.62	59.06
	VEGFA	53.03	62.27	69.93	64.79	54.55
	Cytokine	0 day	1 day	7 days	30 days	180 days
	SLURP1	44.16	40.80	44.30	38.19	43.05
	SLURP1	34.69	38.13	43.24	42.29	53.92
	SLURP1	32.80	48.00	51.23	33.87	31.32
	SLURP1	38.15	42.34	55.10	39.22	39.30
	SLURP1	31.59	44.69	41.19	29.40	44.95
	SLURP1	39.02	39.16	48.73	33.45	44.85
	SLURP1	36.12	36.61	48.67	40.11	46.70
	SLURP1	33.03	48.83	49.09	39.20	44.92
**FS-LASIK**	SLURP1	44.59	48.83	48.51	37.12	50.96
	SLURP1	31.70	42.96	50.14	39.98	46.31
	SLURP1	31.89	41.69	43.58	29.22	39.31
	SLURP1	24.00	47.28	46.69	39.73	45.13
	SLURP1	24.86	45.03	49.81	35.91	42.91
	SLURP1	32.23	44.54	51.97	39.78	35.26
	SLURP1	34.13	44.06	60.94	40.51	44.44
	SLURP1	36.82	33.43	52.07	38.05	39.82
	SLURP1	37.79	43.40	49.62	38.14	42.68

	SLURP1	32.12	43.60	48.61	33.66	51.51
	SLURP1	35.29	45.91	60.53	40.69	38.53
	SLURP1	28.76	40.88	49.80	36.12	49.03
	SLURP1	32.49	35.73	43.51	41.37	39.04
	SLURP1	29.23	46.02	50.64	25.22	43.74
	SLURP1	28.83	51.14	48.68	30.58	42.87
	SLURP1	34.90	44.91	41.60	29.27	33.16
	SLURP1	29.09	44.10	51.01	32.82	43.36
	SLURP1	28.72	50.47	46.81	34.63	46.02
	SLURP1	36.91	49.38	49.45	36.85	47.01
	SLURP1	37.33	39.16	42.99	40.81	48.46
	SLURP1	29.62	45.35	51.45	35.53	43.36
	SLURP1	40.56	41.85	54.21	37.56	44.74
	SLURP1	26.40	43.30	44.98	34.85	37.70
	SLURP1	37.78	52.22	48.97	26.32	43.87
	SLURP1	43.11	46.89	52.94	29.83	46.83
	SLURP1	35.13	46.62	45.02	42.07	49.10
	SLURP1	35.09	40.81	43.56	37.13	40.77
	SLURP1	28.07	42.15	49.59	28.44	40.65
	SLURP1	35.06	47.57	48.13	37.82	46.68
	SLURP1	30.49	49.11	37.78	35.90	42.81
	SLURP1	29.40	47.86	49.89	39.53	40.05
	SLURP1	32.13	47.04	45.32	33.74	38.83
	SLURP1	33.68	42.03	48.98	34.86	51.59
	SLURP1	38.77	40.59	52.81	28.68	44.88
	SLURP1	25.34	39.54	49.95	33.09	48.05
	SLURP1	35.84	53.57	47.53	36.52	44.40
	SLURP1	30.90	38.45	48.40	39.77	47.25
	SLURP1	38.57	43.73	37.15	26.14	46.74
	SLURP1	45.24	50.16	47.28	37.67	43.48
	SLURP1	31.68	44.51	39.44	33.50	44.67
	SLURP1	42.60	45.11	45.43	38.02	47.31
	SLURP1	31.95	54.32	46.82	36.07	46.29

	Cytokine	**0 day**	**1 day**	**7 days**	**30 days**	**180 days**
	TGF-B1	26.14	27.83	24.34	34.18	32.69
	TGF-B1	29.67	24.67	26.91	31.15	27.75
	TGF-B1	26.42	28.54	31.49	37.42	36.64
**SMILE**	TGF-B1	27.64	27.62	26.63	34.69	36.10
	TGF-B1	29.84	36.56	23.50	33.36	33.66
	TGF-B1	31.24	25.39	27.31	25.01	31.87
	TGF-B1	26.05	31.45	34.18	36.22	30.91

	TGF-B1	21.91	28.42	39.31	43.65	29.31
	TGF-B1	33.49	30.68	24.42	26.15	30.67
	TGF-B1	31.88	32.18	32.57	20.46	28.98
	TGF-B1	30.21	26.64	39.40	35.73	33.26
	TGF-B1	30.09	33.46	33.94	30.68	22.12
	TGF-B1	32.58	30.06	34.39	38.04	26.34
	TGF-B1	29.35	36.79	27.44	28.58	24.66
	TGF-B1	26.19	34.43	20.86	40.37	28.13
	TGF-B1	36.77	34.97	37.60	33.22	24.79
	TGF-B1	19.01	31.37	24.62	31.29	25.79
	TGF-B1	36.74	27.29	25.15	28.53	27.43
	TGF-B1	31.84	27.57	24.70	35.79	24.29
	TGF-B1	30.26	31.81	23.62	32.37	23.79
	TGF-B1	24.61	33.22	29.15	26.23	25.00
	TGF-B1	32.09	28.59	38.01	26.54	36.52
	TGF-B1	30.59	34.47	33.54	31.57	30.51
	TGF-B1	28.95	32.05	41.27	29.71	35.21
	TGF-B1	28.45	31.25	32.52	27.06	34.38
	TGF-B1	29.99	37.86	28.59	30.08	19.83
	TGF-B1	30.21	26.60	28.36	36.16	38.79
	TGF-B1	29.65	37.33	26.72	32.74	32.22
	TGF-B1	29.02	35.54	41.35	37.22	32.15
	TGF-B1	35.38	29.58	36.72	38.65	36.16
	TGF-B1	34.54	32.46	41.02	39.05	28.81
	TGF-B1	27.78	27.00	33.23	26.15	25.70
	TGF-B1	35.73	30.65	26.97	29.62	32.18
	TGF-B1	27.19	31.89	28.96	32.08	17.78
	TGF-B1	30.27	32.20	30.07	31.69	24.61
	TGF-B1	36.61	33.33	26.50	31.78	29.30
	TGF-B1	36.06	30.81	20.75	42.16	21.71
	TGF-B1	29.66	21.10	30.04	34.68	40.60
	TGF-B1	28.64	28.00	31.22	25.96	21.48
	TGF-B1	30.24	36.79	23.49	43.50	28.65
	TGF-B1	25.89	28.21	28.60	26.81	41.07
	TGF-B1	23.98	37.47	31.44	37.47	32.39
	TGF-B1	26.01	31.30	25.10	32.12	35.53
	TGF-B1	33.34	39.78	30.44	26.30	33.23
	TGF-B1	26.36	26.88	34.22	36.42	24.63
	TGF-B1	33.05	29.00	39.80	30.87	26.86
	TGF-B1	33.41	30.76	31.84	29.81	23.85
	TGF-B1	28.96	28.96	32.02	37.93	28.38
	TGF-B1	30.69	24.28	36.40	37.63	30.91

	TGF-B1	30.84	35.35	33.37	32.03	25.28
	Cytokine	0 day	1 day	7 days	30 days	180 days
	TGFB2	36.51	36.80	45.20	45.12	40.33
	TGFB2	46.63	42.11	36.69	42.22	35.98
	TGFB2	42.77	35.91	40.24	41.77	40.49
	TGFB2	35.47	36.69	41.93	42.18	35.94
	TGFB2	35.86	37.24	36.04	47.60	35.31
	TGFB2	35.65	39.39	35.71	37.19	32.37
	TGFB2	42.02	33.04	44.01	47.32	38.04
	TGFB2	39.47	26.30	49.76	40.63	40.19
	TGFB2	39.18	41.45	36.50	44.39	50.10
	TGFB2	43.90	29.18	47.18	42.16	44.21
	TGFB2	34.70	35.79	32.13	35.47	38.97
	TGFB2	37.14	35.02	39.83	36.55	40.45
	TGFB2	37.57	39.98	49.29	34.05	41.51
	TGFB2	37.24	36.81	29.73	45.97	42.44
	TGFB2	40.37	35.79	39.97	44.09	45.72
	TGFB2	38.28	33.31	40.67	44.89	41.63
	TGFB2	47.07	30.35	41.96	45.75	41.65
	TGFB2	45.56	32.63	38.92	43.88	31.77
	TGFB2	35.74	36.99	43.84	48.43	40.89
**SMILE**	TGFB2	42.51	39.29	40.85	38.60	42.15
	TGFB2	42.08	42.53	39.80	43.41	39.48
	TGFB2	35.14	32.53	39.67	49.59	38.17
	TGFB2	35.47	33.76	43.15	35.96	34.71
	TGFB2	33.20	34.87	42.15	47.59	32.47
	TGFB2	40.64	38.36	31.21	44.95	35.40
	TGFB2	36.61	43.29	44.12	40.45	40.92
	TGFB2	32.37	37.52	43.21	29.74	37.85
	TGFB2	34.23	35.39	47.58	46.10	40.97
	TGFB2	33.79	38.27	40.92	37.34	39.89
	TGFB2	35.39	38.80	50.33	41.30	28.18
	TGFB2	43.52	29.66	33.96	43.50	41.91
	TGFB2	36.64	36.74	39.07	41.86	40.58
	TGFB2	38.41	37.92	49.22	40.88	43.94
	TGFB2	37.00	41.33	43.61	47.09	42.16
	TGFB2	32.11	33.22	46.51	45.35	46.38
	TGFB2	36.84	41.24	40.97	33.18	47.65
	TGFB2	42.88	40.81	52.24	41.44	44.27
	TGFB2	31.74	37.66	44.81	38.35	30.43
	TGFB2	40.17	36.60	41.59	43.38	45.46
	TGFB2	35.02	31.36	36.35	46.36	42.59

	TGFB2	48.55	36.61	44.92	45.06	28.08
	TGFB2	33.47	32.90	53.72	34.74	36.25
	TGFB2	39.79	41.61	39.98	27.32	39.14
	TGFB2	32.33	36.90	38.66	47.39	31.54
	TGFB2	40.82	35.70	39.78	47.06	47.58
	TGFB2	36.30	37.57	43.61	37.41	37.41
	TGFB2	35.97	39.00	40.15	33.19	43.37
	TGFB2	39.67	38.87	38.61	50.81	39.83
	TGFB2	43.24	24.49	49.55	38.71	37.94
	TGFB2	38.12	36.25	44.58	45.75	45.87
**SMILE**	Cytokine	0 day	1 day	7 days	30 days	180 days
	TGFB3	60.72	52.76	49.37	58.27	47.11
	TGFB3	45.48	56.86	50.75	51.19	53.53
	TGFB3	45.82	51.93	43.77	51.96	49.00
	TGFB3	49.13	46.52	50.64	52.68	52.20
	TGFB3	44.86	52.99	60.24	56.30	45.95
	TGFB3	44.79	52.38	55.28	61.80	58.39
	TGFB3	51.85	51.59	50.12	58.23	54.23
	TGFB3	53.20	57.28	50.42	47.12	47.99
	TGFB3	54.99	51.25	53.71	51.51	50.97
	TGFB3	51.99	55.84	55.80	57.29	48.35
	TGFB3	57.59	60.80	49.73	53.21	48.67
	TGFB3	50.95	56.69	56.65	57.38	49.27
	TGFB3	47.60	52.80	44.20	49.61	54.88
	TGFB3	59.50	58.21	50.06	54.79	49.69
	TGFB3	50.87	59.37	58.45	46.78	55.44
	TGFB3	50.84	51.69	59.16	45.03	48.35
	TGFB3	49.61	56.34	51.32	55.32	57.00
	TGFB3	53.67	41.38	49.17	44.89	50.45
	TGFB3	44.70	45.88	47.26	49.37	58.78
	TGFB3	47.96	51.23	52.95	57.16	51.93
	TGFB3	55.90	54.77	47.45	54.30	55.12
	TGFB3	49.59	56.19	49.60	61.77	50.22
	TGFB3	47.78	57.67	57.58	52.76	53.55
	TGFB3	47.29	60.42	59.60	46.10	52.15
	TGFB3	42.27	61.10	57.35	46.45	55.39
	TGFB3	51.01	66.28	58.93	43.02	53.66
	TGFB3	51.38	58.82	58.52	51.34	55.84
	TGFB3	48.03	52.57	58.25	49.62	53.24
	TGFB3	51.11	45.25	53.33	53.74	50.77
	TGFB3	46.80	59.00	46.78	57.42	49.08
	TGFB3	48.12	49.53	51.62	51.86	52.58

	TGFB3	51.46	60.19	58.39	58.46	59.61
	TGFB3	50.22	49.82	53.66	44.36	52.06
	TGFB3	51.87	55.30	56.25	52.09	65.72
	TGFB3	53.44	56.17	60.65	52.79	51.97
	TGFB3	49.72	61.54	56.29	46.07	46.53
	TGFB3	54.66	56.94	50.79	52.13	55.78
	TGFB3	53.53	49.41	49.77	56.04	49.54
	TGFB3	42.94	62.49	49.36	54.70	52.88
	TGFB3	55.96	60.94	53.02	65.30	49.85
	TGFB3	42.59	48.93	48.08	56.55	50.64
	TGFB3	39.91	64.85	48.89	51.43	50.40
	TGFB3	48.38	63.92	50.40	52.79	55.47
	TGFB3	54.80	60.14	55.34	54.40	50.98
	TGFB3	48.34	53.68	55.97	62.47	46.60
	TGFB3	56.90	47.88	55.96	52.27	56.66
	TGFB3	50.89	60.15	47.11	50.76	51.33
	TGFB3	62.12	51.09	42.28	43.84	41.75
	TGFB3	50.10	54.50	49.37	49.42	51.14
	TGFB3	54.15	61.95	47.80	49.83	58.03
	Cytokine	0 day	1 day	7 days	30 days	180 days
	IL1B	58.49	61.68	62.81	55.06	56.34
	IL1B	66.52	61.74	64.77	67.05	58.81
	IL1B	63.39	69.98	56.37	54.73	58.69
	IL1B	63.64	72.01	58.61	60.81	57.63
	IL1B	61.66	79.61	60.72	69.82	52.74
	IL1B	59.12	67.50	66.29	63.78	63.33
	IL1B	63.67	62.74	64.85	62.14	68.03
	IL1B	62.97	57.83	63.93	67.93	59.53
	IL1B	63.01	63.78	62.99	60.87	57.32
	IL1B	65.41	72.19	64.15	65.48	65.50
**SMILE**	IL1B	66.15	65.10	57.51	54.19	64.44
	IL1B	58.97	59.97	69.22	59.21	66.15
	IL1B	63.07	64.50	70.71	51.48	64.83
	IL1B	55.41	70.60	64.50	61.82	56.99
	IL1B	55.36	62.08	51.75	60.70	61.62
	IL1B	63.31	63.80	63.94	71.04	66.62
	IL1B	55.74	61.80	65.40	68.99	66.90
	IL1B	55.94	58.91	67.88	58.61	49.14
	IL1B	62.45	60.37	61.37	67.11	57.51
	IL1B	78.53	65.29	62.93	58.93	61.70
	IL1B	65.32	58.20	53.27	56.95	66.87
	IL1B	64.18	62.54	54.73	72.99	62.87

	IL1B	64.02	62.55	61.29	62.85	56.95
	IL1B	60.03	52.86	49.00	70.07	61.09
	IL1B	56.17	57.69	67.62	68.16	60.22
	IL1B	67.11	61.23	64.51	62.39	57.87
	IL1B	62.78	56.36	62.17	67.18	61.60
	IL1B	58.82	67.38	53.38	74.21	68.57
	IL1B	68.47	63.33	59.47	63.94	68.23
	IL1B	71.88	61.06	64.87	65.54	59.91
	IL1B	67.50	53.87	54.14	59.35	59.02
	IL1B	59.24	62.30	67.87	53.35	64.40
	IL1B	74.02	62.60	58.64	66.24	61.03
	IL1B	72.63	44.69	69.16	66.14	60.63
	IL1B	65.16	66.46	58.75	64.39	66.42
	IL1B	62.16	62.90	60.96	62.94	55.20
	IL1B	58.10	75.37	56.63	68.50	65.58
	IL1B	67.20	58.11	53.92	61.36	52.22
	IL1B	68.12	64.96	54.18	63.83	66.19
	IL1B	56.45	61.83	67.88	62.03	63.40
	IL1B	56.43	66.69	62.57	65.12	60.99
	IL1B	55.11	58.24	52.71	66.97	65.22
	IL1B	62.17	59.92	56.43	69.94	64.21
	IL1B	54.73	63.82	57.82	61.29	59.96
	IL1B	60.25	61.52	67.03	60.03	53.75
	IL1B	62.22	66.16	62.42	61.16	67.76
	IL1B	51.66	53.99	64.89	75.02	63.00
	IL1B	64.46	58.50	57.64	70.58	58.01
	IL1B	51.27	54.39	57.88	61.17	62.31
	IL1B	67.90	71.23	58.79	67.19	60.34
**SMILE**	Cytokine	0 day	1 day	7 days	30 days	180 days
	IL15	11.08	14.66	27.08	21.45	10.39
	IL15	13.28	21.04	18.88	22.30	16.63
	IL15	9.44	25.79	23.80	18.67	12.10
	IL15	15.42	23.52	25.08	21.83	20.62
	IL15	13.54	13.43	20.84	16.92	5.53
	IL15	1.02	26.00	18.38	29.19	9.85
	IL15	4.48	9.35	15.45	27.09	21.57
	IL15	12.43	26.24	31.23	19.58	6.84
	IL15	7.31	23.45	22.80	17.96	12.79
	IL15	7.43	12.60	22.93	16.47	15.65
	IL15	23.84	24.96	23.81	15.02	15.73
	IL15	3.17	24.04	29.30	15.41	9.77
	IL15	10.53	17.26	20.38	23.72	16.26

	IL15	10.76	20.44	25.08	15.16	15.17
	IL15	10.69	18.48	25.83	29.61	10.70
	IL15	4.09	22.91	19.18	20.63	14.17
	IL15	12.58	25.98	22.14	24.09	14.20
	IL15	13.98	29.21	5.63	17.09	3.91
	IL15	7.70	28.00	18.14	25.68	13.48
	IL15	13.78	23.82	23.63	28.38	11.25
	IL15	10.17	23.70	17.54	21.50	11.35
	IL15	23.33	24.24	22.42	21.68	14.73
	IL15	7.17	26.55	22.01	25.61	9.28
	IL15	13.26	32.34	17.95	21.95	8.08
	IL15	15.12	21.41	25.53	26.84	11.44
	IL15	21.20	30.17	22.82	35.40	9.73
	IL15	11.12	19.78	27.37	26.15	14.06
	IL15	10.71	19.49	22.00	24.33	10.94
	IL15	19.40	15.73	21.14	24.81	14.37
	IL15	16.71	24.37	22.32	20.70	9.09
	IL15	-0.61	23.63	22.02	13.89	13.86
	IL15	15.67	16.66	18.86	22.81	11.71
	IL15	15.08	22.99	21.55	17.91	14.47
	IL15	13.62	23.18	26.96	29.52	14.22
	IL15	11.22	29.73	29.39	29.03	7.23
	IL15	3.67	17.27	13.50	30.78	8.20
	IL15	13.43	18.19	21.49	24.72	15.35
	IL15	7.26	21.87	21.71	13.36	13.81
	IL15	13.43	19.24	28.45	17.07	21.71
	IL15	9.15	24.56	22.66	23.17	9.39
	IL15	19.17	26.30	16.74	20.91	11.60
	IL15	18.77	21.26	26.97	16.94	5.42
	IL15	2.89	21.52	21.61	16.66	9.16
	IL15	8.09	17.49	19.94	22.35	14.35
	IL15	9.91	25.80	26.32	21.31	12.33
	IL15	9.40	23.29	16.78	38.47	15.14
	IL15	10.54	21.29	20.54	38.85	9.42
	IL15	17.33	19.06	17.07	21.66	19.55
	IL15	10.27	24.87	17.78	20.16	18.16
	IL15	10.38	23.65	18.76	17.91	8.96
**SMILE**	Cytokine	0 day	1 day	7 days	30 days	180 days
	IHNB	9.74	19.95	15.23	12.19	12.24
	IHNB	11.90	15.50	18.51	5.79	19.49
	IHNB	26.39	20.55	8.48	17.93	17.55
	IHNB	11.04	13.07	10.88	11.47	24.25

	IHNB	10.45	13.58	14.26	13.29	8.41
	IHNB	12.51	17.28	6.96	5.82	16.26
	IHNB	12.39	18.43	16.94	20.19	3.89
	IHNB	7.76	5.31	4.85	7.14	4.60
	IHNB	14.61	11.22	16.02	21.55	17.86
	IHNB	15.39	21.68	19.69	9.11	12.38
	IHNB	13.82	14.60	11.35	7.65	18.78
	IHNB	18.16	6.67	10.42	18.85	13.93
	IHNB	4.97	14.54	15.61	-3.89	9.51
	IHNB	13.88	9.65	19.84	10.98	15.77
	IHNB	11.92	13.72	11.10	23.95	12.88
	IHNB	11.75	9.80	9.04	23.57	14.89
	IHNB	15.79	10.29	12.17	3.76	12.36
	IHNB	11.83	16.22	12.13	12.81	18.92
	IHNB	5.28	23.10	12.62	12.64	14.03
	IHNB	16.77	15.82	16.62	13.06	21.51
	IHNB	9.78	9.31	18.71	8.28	14.43
	IHNB	21.77	22.92	12.75	15.63	19.23
	IHNB	19.02	3.83	5.64	6.38	6.91
	IHNB	6.59	9.00	13.32	12.86	8.64
	IHNB	17.34	18.90	3.40	18.72	12.32
	IHNB	9.67	9.53	16.00	16.84	14.44
	IHNB	8.83	14.54	3.99	5.99	4.51
	IHNB	16.88	17.64	17.43	10.41	23.00
	IHNB	11.73	16.51	25.96	16.27	15.63
	IHNB	9.07	9.96	16.34	15.39	15.54
	IHNB	24.78	14.30	20.48	17.92	8.60
	IHNB	4.17	19.54	12.49	14.97	15.36
	IHNB	10.28	17.09	11.57	19.81	10.58
	IHNB	16.98	1.89	7.82	19.57	15.58
	IHNB	22.46	14.08	6.19	18.61	17.01
	IHNB	10.34	9.22	3.19	13.52	15.51
	IHNB	12.20	3.73	15.83	5.74	16.51
	IHNB	11.32	15.41	15.67	14.11	6.98
	IHNB	11.29	15.81	10.17	15.79	6.94
	IHNB	13.61	19.52	7.01	13.13	3.89
	IHNB	12.96	13.05	7.93	27.93	9.40
	IHNB	8.59	21.37	14.86	9.27	3.90
	IHNB	10.39	12.49	18.69	18.35	15.57
	IHNB	13.34	16.17	14.33	16.57	15.94
	IHNB	12.57	8.31	11.01	12.55	15.50
	IHNB	17.94	21.58	14.66	4.48	22.10

	IHNB	23.59	12.51	16.92	9.83	10.60
	IHNB	17.76	7.37	19.86	11.00	5.36
	IHNB	14.76	8.90	4.29	13.47	22.90
	IHNB	16.66	16.29	8.97	17.02	9.22
SMILE	Cytokine	0 day	1 day	7 days	30 days	180 days
	VEGFA	56.86	77.19	69.42	58.75	60.35
	VEGFA	50.23	81.93	66.06	56.07	56.26
	VEGFA	50.23	80.34	72.74	54.01	57.86
	VEGFA	55.36	80.69	73.24	67.37	57.59
	VEGFA	57.72	81.49	72.95	60.48	66.51
	VEGFA	56.79	75.48	66.60	67.31	61.29
	VEGFA	47.50	83.17	76.16	61.06	58.40
	VEGFA	51.34	78.90	77.47	61.26	55.33
	VEGFA	61.19	76.17	72.48	61.89	59.90
	VEGFA	51.84	80.99	71.55	65.68	57.90
	VEGFA	53.98	85.66	69.87	66.99	63.07
	VEGFA	48.71	66.20	72.47	68.83	59.75
	VEGFA	56.45	75.38	64.82	60.29	59.36
	VEGFA	59.84	70.27	68.51	53.73	60.85
	VEGFA	51.09	83.21	75.78	64.30	64.14
	VEGFA	65.10	73.32	64.87	69.42	55.01
	VEGFA	48.60	81.95	76.85	56.19	60.45
	VEGFA	57.74	71.33	69.07	60.41	58.23
	VEGFA	64.10	81.84	74.47	66.99	62.91
	VEGFA	49.73	74.27	75.03	56.52	66.34
	VEGFA	39.73	77.33	71.61	56.41	65.18
	VEGFA	47.71	80.41	72.66	56.23	59.76
	VEGFA	56.38	69.51	57.32	55.84	49.90
	VEGFA	57.63	80.03	75.75	54.90	57.24
	VEGFA	54.52	91.37	80.26	62.06	62.06
	VEGFA	60.94	75.40	80.21	60.27	57.83
	VEGFA	49.55	84.04	71.39	63.34	62.36
	VEGFA	52.23	77.86	76.69	55.33	66.49
	VEGFA	56.54	83.11	75.13	55.44	56.95
	VEGFA	45.67	77.66	68.65	62.95	58.58
	VEGFA	52.54	78.75	75.38	60.29	49.06
	VEGFA	58.89	76.98	74.73	66.44	64.59
	VEGFA	49.29	83.33	61.61	61.28	63.77
	VEGFA	59.73	76.61	65.23	64.05	60.70
	VEGFA	53.89	73.10	64.70	69.88	64.15
	VEGFA	57.65	72.68	68.40	53.49	57.76
	VEGFA	56.14	75.40	72.32	55.96	50.24

	VEGFA	62.85	77.40	69.30	55.03	67.57
	VEGFA	49.78	80.10	74.75	55.67	58.48
	VEGFA	57.44	68.81	72.90	62.73	55.15
	VEGFA	50.37	87.94	73.16	59.61	55.14
	VEGFA	54.34	80.38	72.01	59.03	57.99
	VEGFA	55.39	83.41	74.53	64.55	61.09
	VEGFA	51.84	80.69	68.59	65.63	60.15
	VEGFA	54.92	76.47	69.78	69.78	65.37
	VEGFA	49.75	80.94	74.89	54.87	59.23
	VEGFA	55.65	80.36	83.23	53.84	59.02
	VEGFA	55.50	80.77	70.68	58.34	49.41
	VEGFA	51.14	80.00	74.50	60.48	50.33
	VEGFA	57.38	76.73	76.16	62.66	61.58
	Cytokine	0 day	1 day	7 days	30 days	180 days
**SMILE**	SLURP1	31.00	90.14	37.80	37.21	44.35
	SLURP1	36.01	96.64	35.34	34.14	24.05
	SLURP1	38.92	91.53	34.50	35.32	32.40
	SLURP1	42.61	95.10	34.33	35.78	39.01
	SLURP1	42.23	102.20	38.22	40.14	35.84
	SLURP1	27.14	103.64	38.63	42.40	31.95
	SLURP1	46.31	102.56	38.76	37.88	43.85
	SLURP1	37.60	93.06	33.82	33.07	42.23
	SLURP1	42.21	101.43	42.33	40.73	39.17
	SLURP1	32.23	96.99	45.08	38.10	33.59
	SLURP1	28.75	93.27	38.06	42.08	34.65
	SLURP1	39.14	97.24	37.20	33.22	36.61
	SLURP1	30.89	101.10	34.06	48.90	36.40
	SLURP1	38.38	96.31	46.02	42.74	43.25
	SLURP1	34.97	98.65	42.23	37.86	27.30
	SLURP1	42.54	94.85	34.99	43.70	41.55
	SLURP1	42.42	84.76	38.57	37.90	37.22
	SLURP1	29.31	93.30	39.01	44.91	39.58
	SLURP1	36.30	98.28	32.04	38.52	40.18
	SLURP1	40.40	96.97	40.80	36.13	38.44
	SLURP1	37.69	93.61	37.29	30.00	26.93
	SLURP1	34.76	97.28	37.00	33.71	30.36
	SLURP1	27.79	90.20	36.94	42.90	42.97
	SLURP1	37.04	93.91	39.20	36.71	33.43
	SLURP1	29.12	88.25	35.15	42.41	35.59
	SLURP1	33.61	84.77	36.05	41.22	32.34
	SLURP1	34.91	94.46	40.18	34.64	40.82
	SLURP1	40.12	106.78	42.31	38.53	39.82

	SLURP1	26.75	98.22	31.63	36.73	36.85
	SLURP1	40.45	94.99	33.85	40.43	40.49
	SLURP1	49.77	97.99	37.18	41.31	33.26
	SLURP1	36.09	106.73	41.71	30.69	41.70
	SLURP1	39.08	93.37	36.42	41.06	35.36
	SLURP1	31.58	92.46	31.74	35.82	39.68
	SLURP1	30.54	99.85	31.64	38.98	30.64
	SLURP1	39.84	101.97	42.70	39.70	29.88
	SLURP1	33.50	101.84	52.42	33.81	35.65
	SLURP1	34.73	88.32	42.72	28.35	35.98
	SLURP1	32.57	97.71	49.83	36.65	35.95
	SLURP1	41.99	96.88	42.18	42.33	35.51
	SLURP1	30.34	91.94	38.17	43.44	38.37
	SLURP1	30.71	103.16	38.74	36.58	29.37
	SLURP1	35.84	99.64	40.74	38.71	44.61
	SLURP1	33.45	103.51	34.09	32.06	36.20
	SLURP1	33.31	91.97	51.31	34.16	36.91
	SLURP1	32.63	93.68	40.29	33.36	49.95
	SLURP1	42.10	92.92	30.30	33.00	34.61
	SLURP1	29.49	97.29	40.75	34.94	35.49
	SLURP1	32.09	99.20	39.72	39.13	39.05
	SLURP1	33.23	91.40	44.18	47.29	41.85

TGF-β*1-3*, transforming growth factor beta 1-3; *IL-1B*- interleukina 1 beta; IL-15, interleukin 15; IHNBA, inhibin beta A chain; VEGG, vascular endothelial growth factor A; SLURP1, secreted Ly-6_uPAR-related protein 1; PRK, photorefractive keratectomy;
FS-LASIK, femtosecond-assisted laser in-situ keratomileusis; SMILE, refractive lenticule extraction with small incision lenticule extraction
