# Supplementary material for: Comparative Analysis of Corneal Wound Healing: Differential Molecular Responses in Tears Following PRK, FS-LASIK, and SMILE Procedures
Source: Biomedicines. 2024 Oct 9;12(10):2289. doi: 10.3390/biomedicines12102289 (PMC11505177; doi:10.3390/biomedicines12102289)
Supplement: Supplementary file 1 [file biomedicines-12-02289-s001.zip › Table S2.pdf]

Table S2: The detailed results of the post hoc test of the individual comparisons of the concentrations of a given cytokine at follow-up depending on the LVC used.

Group vs. Group (Contrast)	PRK	FS-LASIK	SMILE
		p-value	
0 day IHNB vs 0 day IL15	1	1	1
0 day IHNB vs 0 day IL1B	0	0	0
0 day IHNB vs 0 day SLURP1	0	0	0
0 day IHNB vs 0 day TGFB1	0	0	0
0 day IHNB vs 0 day TGFB2	0	0	0
0 day IHNB vs 0 day TGFB3	0	0	0
0 day IHNB vs 0 day VEGFA	0	0	0
0 day IHNB vs 1 day IHNB	1	0	1
0 day IHNB vs 1 day IL15	0.76616	0	0.00033
0 day IHNB vs 1 day IL1B	0	0	0
0 day IHNB vs 1 day SLURP1	0	0	0
0 day IHNB vs 1 day TGFB1	0	0	0
0 day IHNB vs 1 day TGFB2	0	0	0
0 day IHNB vs 1 day TGFB3	0	0	0
0 day IHNB vs 1 day VEGFA	0	0	0
0 day IHNB vs 180 days	0	0	0
0 day IHNB vs 180 days IHNB	1	0.89877	1
0 day IHNB vs 180 days IL15	1	1.39E-12	1
0 day IHNB vs 180 days SLURP1	0	0	0
0 day IHNB vs 180 days TGFB1	0	0	0
0 day IHNB vs 180 days TGFB2	0	0	0
0 day IHNB vs 180 days TGFB3	0	0	0
0 day IHNB vs 180 days VEGFA	0	0	0
0 day IHNB vs 30 days IHNB	1	1.64E-09	1
0 day IHNB vs 30 days IL15	1	0	0.00004
0 day IHNB vs 30 days IL1B	0	0	0
0 day IHNB vs 30 days SLURP1	0	0	0
0 day IHNB vs 30 days TGFB1	0	0	0
0 day IHNB vs 30 days TGFB2	0	0	0
0 day IHNB vs 30 days TGFB3	0	0	0
0 day IHNB vs 30 days VEGFA	0	0	0
0 day IHNB vs 7 days IHNB	1	0	1
0 day IHNB vs 7 days IL15	0.99998	0	0.00202
0 day IHNB vs 7 days IL1B	0	0	0

0 day IHNB vs 7 days SLURP1	0	0	0
0 day IHNB vs 7 days TGFB1	0	0	0
0 day IHNB vs 7 days TGFB2	0	0	0
0 day IHNB vs 7 days TGFB3	0	0	0
0 day IHNB vs 7 days VEGFA	0	0	0
0 day IL15 vs 0 day IL1B	0	0	0
0 day IL15 vs 0 day SLURP1	0	0	0
0 day IL15 vs 0 day TGFB1	0	0	0
0 day IL15 vs 0 day TGFB2	0	0	0
0 day IL15 vs 0 day TGFB3	0	0	0
0 day IL15 vs 0 day VEGFA	0	0	0
0 day IL15 vs 1 day IHNB	1	0	1
0 day IL15 vs 1 day IL15	0.73022	0	2.99E-09
0 day IL15 vs 1 day IL1B	0	0	0
0 day IL15 vs 1 day SLURP1	0	0	0
0 day IL15 vs 1 day TGFB1	0	0	0
0 day IL15 vs 1 day TGFB2	0	0	0
0 day IL15 vs 1 day TGFB3	0	0	0
0 day IL15 vs 1 day VEGFA	0	0	0
0 day IL15 vs 180 days	0	0	0
0 day IL15 vs 180 days IHNB	1	0.03907	1
0 day IL15 vs 180 days IL15	1	0	1
0 day IL15 vs 180 days SLURP1	0	0	0
0 day IL15 vs 180 days TGFB1	0	0	0
0 day IL15 vs 180 days TGFB2	0	0	0
0 day IL15 vs 180 days TGFB3	0	0	0
0 day IL15 vs 180 days VEGFA	0	0	0
0 day IL15 vs 30 days IHNB	1	0	1
0 day IL15 vs 30 days IL15	1	0	1.24E-10
0 day IL15 vs 30 days IL1B	0	0	0
0 day IL15 vs 30 days SLURP1	0	0	0
0 day IL15 vs 30 days TGFB1	0	0	0
0 day IL15 vs 30 days TGFB2	0	0	0
0 day IL15 vs 30 days TGFB3	0	0	0
0 day IL15 vs 30 days VEGFA	0	0	0
0 day IL15 vs 7 days IHNB	1	0	1
0 day IL15 vs 7 days IL15	0.99997	0	5.18E-08
0 day IL15 vs 7 days IL1B	0	0	0
0 day IL15 vs 7 days SLURP1	0	0	0
0 day IL15 vs 7 days TGFB1	0	0	0
0 day IL15 vs 7 days TGFB2	0	0	0
0 day IL15 vs 7 days TGFB3	0	0	0

0 day IL15 vs 7 days VEGFA	0	0	0
0 day IL1B vs 0 day SLURP1	0	0	0
0 day IL1B vs 0 day TGFB1	0	0	0
0 day IL1B vs 0 day TGFB2	1.98E-11	0.86235	0
0 day IL1B vs 0 day TGFB3	0	0	0
0 day IL1B vs 0 day VEGFA	1	0.99998	0.00383
0 day IL1B vs 1 day IHNB	0	0	0
0 day IL1B vs 1 day IL15	0	0	0
0 day IL1B vs 1 day IL1B	0.99998	1	1
0 day IL1B vs 1 day SLURP1	0	0	0
0 day IL1B vs 1 day TGFB1	0.10438	0	0
0 day IL1B vs 1 day TGFB2	0	0	0
0 day IL1B vs 1 day TGFB3	0.97485	0	0.11244
0 day IL1B vs 1 day VEGFA	0	1	0
0 day IL1B vs 180 days	0.25433	1	1
0 day IL1B vs 180 days IHNB	0	0	0
0 day IL1B vs 180 days IL15	0	0	0
0 day IL1B vs 180 days SLURP1	0	0	0
0 day IL1B vs 180 days TGFB1	0	0	0
0 day IL1B vs 180 days TGFB2	3.95E-09	1	0
0 day IL1B vs 180 days TGFB3	0	1.70E-08	1.59E-07
0 day IL1B vs 180 days VEGFA	1	0.05165	1
0 day IL1B vs 30 days IHNB	0	0	0
0 day IL1B vs 30 days IL15	0	0	0
0 day IL1B vs 30 days IL1B	0	0.99409	1
0 day IL1B vs 30 days SLURP1	0	0	0
0 day IL1B vs 30 days TGFB1	0.02769	0.05556	0
0 day IL1B vs 30 days TGFB2	0.99997	0	0
0 day IL1B vs 30 days TGFB3	0.00516	0.97062	2.65E-06
0 day IL1B vs 30 days VEGFA	0	0.93686	1
0 day IL1B vs 7 days IHNB	0	1	0
0 day IL1B vs 7 days IL15	0	1.45E-12	0
0 day IL1B vs 7 days IL1B	0.10699	0.99984	1
0 day IL1B vs 7 days SLURP1	0	0	0
0 day IL1B vs 7 days TGFB1	1	0	0
0 day IL1B vs 7 days TGFB2	1	0	0
0 day IL1B vs 7 days TGFB3	0.00908	0.91783	1.24E-06
0 day IL1B vs 7 days VEGFA	0	1	5.16E-06
0 day SLURP1 vs 0 day TGFB1	0.98296	0.13622	0.71442
0 day SLURP1 vs 0 day TGFB2	0.1357	0	1
0 day SLURP1 vs 0 day TGFB3	0.99941	0	0
0 day SLURP1 vs 0 day VEGFA	0	0	0

0 day SLURP1 vs 1 day IHNB	0	0	0
0 day SLURP1 vs 1 day IL15	0.00017	0.19258	0
0 day SLURP1 vs 1 day IL1B	0	0	0
0 day SLURP1 vs 1 day SLURP1	1	5.19E-08	0
0 day SLURP1 vs 1 day TGFB1	0	0	0.98654
0 day SLURP1 vs 1 day TGFB2	0	0	1
0 day SLURP1 vs 1 day TGFB3	0	0	0
0 day SLURP1 vs 1 day VEGFA	0	0	0
0 day SLURP1 vs 180 days	1.71E-12	0	0
0 day SLURP1 vs 180 days IHNB	0	0	0
0 day SLURP1 vs 180 days IL15	0	0.00446	0
0 day SLURP1 vs 180 days SLURP1	1	1.15E-06	1
0 day SLURP1 vs 180 days TGFB1	0.99962	0	0.4697
0 day SLURP1 vs 180 days TGFB2	0.01849	0	0.99971
0 day SLURP1 vs 180 days TGFB3	0.36348	0	0
0 day SLURP1 vs 180 days VEGFA	0	0	0
0 day SLURP1 vs 30 days IHNB	0	0.00008	0
0 day SLURP1 vs 30 days IL15	0	0.99999	0
0 day SLURP1 vs 30 days IL1B	1	0	0
0 day SLURP1 vs 30 days SLURP1	1	1	1
0 day SLURP1 vs 30 days TGFB1	1.62E-09	0	1
0 day SLURP1 vs 30 days TGFB2	0	0	0.49812
0 day SLURP1 vs 30 days TGFB3	4.55E-08	0	0
0 day SLURP1 vs 30 days VEGFA	0	0	0
0 day SLURP1 vs 7 days IHNB	0	0	0
0 day SLURP1 vs 7 days IL15	6.18E-10	0	0
0 day SLURP1 vs 7 days IL1B	0	0	0
0 day SLURP1 vs 7 days SLURP1	1	0	1
0 day SLURP1 vs 7 days TGFB1	0	0	0.95619
0 day SLURP1 vs 7 days TGFB2	0	0	0.48736
0 day SLURP1 vs 7 days TGFB3	0	0	0
0 day SLURP1 vs 7 days VEGFA	0	0	0
0 day TGFB1 vs 0 day TGFB2	1	0	0.0016
0 day TGFB1 vs 0 day TGFB3	1	0.41182	0
0 day TGFB1 vs 0 day VEGFA	0	0	0
0 day TGFB1 vs 1 day IHNB	0	0	0
0 day TGFB1 vs 1 day IL15	0	0	0.02021
0 day TGFB1 vs 1 day IL1B	0	0	0
0 day TGFB1 vs 1 day SLURP1	0.18417	1	0
0 day TGFB1 vs 1 day TGFB1	0	0	1
0 day TGFB1 vs 1 day TGFB2	0	0	0.37457
0 day TGFB1 vs 1 day TGFB3	0.00011	0	0

0 day TGFB1 vs 1 day VEGFA	0	0	0
0 day TGFB1 vs 180 days	0.07776	0	0
0 day TGFB1 vs 180 days IHNB	0	0	0
0 day TGFB1 vs 180 days IL15	0	0	0
0 day TGFB1 vs 180 days SLURP1	0.99805	1	0.15494
0 day TGFB1 vs 180 days TGFB1	1	0.08148	1
0 day TGFB1 vs 180 days TGFB2	1	0	2.46E-06
0 day TGFB1 vs 180 days TGFB3	1	0.00081	0
0 day TGFB1 vs 180 days VEGFA	0	2.69E-12	0
0 day TGFB1 vs 30 days IHNB	0	0	0
0 day TGFB1 vs 30 days IL15	0	2.27E-08	0.07034
0 day TGFB1 vs 30 days IL1B	1	0	0
0 day TGFB1 vs 30 days SLURP1	0.88493	0.87147	0.00546
0 day TGFB1 vs 30 days TGFB1	0.44516	0	1
0 day TGFB1 vs 30 days TGFB2	1.35E-07	0	0
0 day TGFB1 vs 30 days TGFB3	0.72033	0	0
0 day TGFB1 vs 30 days VEGFA	0	0	0
0 day TGFB1 vs 7 days IHNB	0	0	0
0 day TGFB1 vs 7 days IL15	0	0.06316	0.00476
0 day TGFB1 vs 7 days IL1B	0	0	0
0 day TGFB1 vs 7 days SLURP1	0.30598	0.16071	0.00021
0 day TGFB1 vs 7 days TGFB1	0	0	1
0 day TGFB1 vs 7 days TGFB2	0	0	0
0 day TGFB1 vs 7 days TGFB3	0	0	0
0 day TGFB1 vs 7 days VEGFA	0	0	0
0 day TGFB2 vs 0 day TGFB3	1	0.06377	0
0 day TGFB2 vs 0 day VEGFA	0	1	0
0 day TGFB2 vs 1 day IHNB	0	0	0
0 day TGFB2 vs 1 day IL15	0	0	0
0 day TGFB2 vs 1 day IL1B	0	0.99995	0
0 day TGFB2 vs 1 day SLURP1	0.00009	7.10E-08	0
0 day TGFB2 vs 1 day TGFB1	0	0	0.06379
0 day TGFB2 vs 1 day TGFB2	0	0	1
0 day TGFB2 vs 1 day TGFB3	0.19824	0	0
0 day TGFB2 vs 1 day VEGFA	0	0.70481	0
0 day TGFB2 vs 180 days	0.95947	1	0
0 day TGFB2 vs 180 days IHNB	0	0	0
0 day TGFB2 vs 180 days IL15	0	0	0
0 day TGFB2 vs 180 days SLURP1	0.32542	2.46E-09	1
0 day TGFB2 vs 180 days TGFB1	1	0.36045	0.00022
0 day TGFB2 vs 180 days TGFB2	1	0.23411	1
0 day TGFB2 vs 180 days TGFB3	1	0.94579	0

0 day TGFB2 vs 180 days VEGFA	0	1	0
0 day TGFB2 vs 30 days IHNB	0	0	0
0 day TGFB2 vs 30 days IL15	0	0	0
0 day TGFB2 vs 30 days IL1B	0.99813	1	0
0 day TGFB2 vs 30 days SLURP1	0.02932	0	1
0 day TGFB2 vs 30 days TGFB1	0.99945	0	0.80994
0 day TGFB2 vs 30 days TGFB2	0.00712	0	0.99998
0 day TGFB2 vs 30 days TGFB3	0.99998	1	0
0 day TGFB2 vs 30 days VEGFA	0	1	0
0 day TGFB2 vs 7 days IHNB	0	0.10362	0
0 day TGFB2 vs 7 days IL15	0	0.41385	0
0 day TGFB2 vs 7 days IL1B	0	1	0
0 day TGFB2 vs 7 days SLURP1	0.00034	0.22051	1
0 day TGFB2 vs 7 days TGFB1	0	0	0.02532
0 day TGFB2 vs 7 days TGFB2	0	0	0.99997
0 day TGFB2 vs 7 days TGFB3	0	6.45E-09	0
0 day TGFB2 vs 7 days VEGFA	0	0.99892	0
0 day TGFB3 vs 0 day VEGFA	0	0.00005	0.99995
0 day TGFB3 vs 1 day IHNB	0	0	0
0 day TGFB3 vs 1 day IL15	0	0	0
0 day TGFB3 vs 1 day IL1B	0	6.76E-10	0
0 day TGFB3 vs 1 day SLURP1	0.5215	1	0
0 day TGFB3 vs 1 day TGFB1	0	0	0
0 day TGFB3 vs 1 day TGFB2	0	0	0
0 day TGFB3 vs 1 day TGFB3	3.05E-06	0	0.98179
0 day TGFB3 vs 1 day VEGFA	0	0	0
0 day TGFB3 vs 180 days	0.01024	4.71E-07	2.00E-09
0 day TGFB3 vs 180 days IHNB	0	0	0
0 day TGFB3 vs 180 days IL15	0	0	0
0 day TGFB3 vs 180 days SLURP1	0.99998	0.99999	0
0 day TGFB3 vs 180 days TGFB1	1	1	0
0 day TGFB3 vs 180 days TGFB2	0.99991	0	7.79E-10
0 day TGFB3 vs 180 days TGFB3	1	1	1
0 day TGFB3 vs 180 days VEGFA	0	0.88658	0.00024
0 day TGFB3 vs 30 days IHNB	0	0	0
0 day TGFB3 vs 30 days IL15	0	0	0
0 day TGFB3 vs 30 days IL1B	1	0.00311	0
0 day TGFB3 vs 30 days SLURP1	0.98816	1.78E-12	0
0 day TGFB3 vs 30 days TGFB1	0.1393	0	0
0 day TGFB3 vs 30 days TGFB2	1.53E-09	0	0.00047
0 day TGFB3 vs 30 days TGFB3	0.34727	0.01322	1
0 day TGFB3 vs 30 days VEGFA	0	0.02801	1.83E-07

0 day TGFB3 vs 7 days IHNB	0	0	0
0 day TGFB3 vs 7 days IL15	0	1	0
0 day TGFB3 vs 7 days IL1B	0	0.00018	1.90E-08
0 day TGFB3 vs 7 days SLURP1	0.67864	1	1.13E-12
0 day TGFB3 vs 7 days TGFB1	0	0	0
0 day TGFB3 vs 7 days TGFB2	0	0	0.00052
0 day TGFB3 vs 7 days TGFB3	0	0	1
0 day TGFB3 vs 7 days VEGFA	0	1.74E-11	0
0 day VEGFA vs 1 day IHNB	0	0	0
0 day VEGFA vs 1 day IL15	0	0	0
0 day VEGFA vs 1 day IL1B	1	1	0.00127
0 day VEGFA vs 1 day SLURP1	0	0	0
0 day VEGFA vs 1 day TGFB1	0.99318	0	0
0 day VEGFA vs 1 day TGFB2	5.64E-09	0	0
0 day VEGFA vs 1 day TGFB3	0.04629	0	1
0 day VEGFA vs 1 day VEGFA	0	0.9997	0
0 day VEGFA vs 180 days	0.00004	1	0.0899
0 day VEGFA vs 180 days IHNB	0	0	0
0 day VEGFA vs 180 days IL15	0	0	0
0 day VEGFA vs 180 days SLURP1	0	0	0
0 day VEGFA vs 180 days TGFB1	0	0.00232	0
0 day VEGFA vs 180 days TGFB2	0	0.97592	0
0 day VEGFA vs 180 days TGFB3	0	0.14582	1
0 day VEGFA vs 180 days VEGFA	1	0.99985	0.92677
0 day VEGFA vs 30 days IHNB	0	0	0
0 day VEGFA vs 30 days IL15	0	0	0
0 day VEGFA vs 30 days IL1B	0	1	3.98E-06
0 day VEGFA vs 30 days SLURP1	0	0	0
0 day VEGFA vs 30 days TGFB1	2.21E-07	1.03E-09	0
0 day VEGFA vs 30 days TGFB2	0.49027	0	0
0 day VEGFA vs 30 days TGFB3	8.99E-09	1	1
0 day VEGFA vs 30 days VEGFA	0	1	0.33511
0 day VEGFA vs 7 days IHNB	0	0.91525	0
0 day VEGFA vs 7 days IL15	0	0.00343	0
0 day VEGFA vs 7 days IL1B	0.9935	1	0.18352
0 day VEGFA vs 7 days SLURP1	0	0.00066	0
0 day VEGFA vs 7 days TGFB1	1	0	0
0 day VEGFA vs 7 days TGFB2	1	0	0
0 day VEGFA vs 7 days TGFB3	0.87583	0.00031	1
0 day VEGFA vs 7 days VEGFA	0	1	0
1 day IHNB vs 1 day IL15	0.36459	0	0.00168
1 day IHNB vs 1 day IL1B	0	0	0

1 day IHNB vs 1 day SLURP1	0	0	0
1 day IHNB vs 1 day TGFB1	0	0	0
1 day IHNB vs 1 day TGFB2	0	0	0
1 day IHNB vs 1 day TGFB3	0	0	0
1 day IHNB vs 1 day VEGFA	0	0	0
1 day IHNB vs 180 days	0	0	0
1 day IHNB vs 180 days IHNB	1	0	1
1 day IHNB vs 180 days IL15	1	0	1
1 day IHNB vs 180 days SLURP1	0	0	0
1 day IHNB vs 180 days TGFB1	0	0	0
1 day IHNB vs 180 days TGFB2	0	0	0
1 day IHNB vs 180 days TGFB3	0	0	0
1 day IHNB vs 180 days VEGFA	0	0	0
1 day IHNB vs 30 days IHNB	1	0	1
1 day IHNB vs 30 days IL15	1	0	0.00025
1 day IHNB vs 30 days IL1B	0	0	0
1 day IHNB vs 30 days SLURP1	0	0	0
1 day IHNB vs 30 days TGFB1	0	0.0076	0
1 day IHNB vs 30 days TGFB2	0	1.92E-11	0
1 day IHNB vs 30 days TGFB3	0	0	0
1 day IHNB vs 30 days VEGFA	0	0	0
1 day IHNB vs 7 days IHNB	1	0	1
1 day IHNB vs 7 days IL15	0.99793	0	0.0083
1 day IHNB vs 7 days IL1B	0	0	0
1 day IHNB vs 7 days SLURP1	0	0	0
1 day IHNB vs 7 days TGFB1	0	1.07E-06	0
1 day IHNB vs 7 days TGFB2	0	0	0
1 day IHNB vs 7 days TGFB3	0	2.03E-07	0
1 day IHNB vs 7 days VEGFA	0	0	0
1 day IL15 vs 1 day IL1B	0	0	0
1 day IL15 vs 1 day SLURP1	0.17883	0	0
1 day IL15 vs 1 day TGFB1	0	0	0.00028
1 day IL15 vs 1 day TGFB2	0	0	0
1 day IL15 vs 1 day TGFB3	0	0	0
1 day IL15 vs 1 day VEGFA	0	0	0
1 day IL15 vs 180 days	0	0	0
1 day IL15 vs 180 days IHNB	0.21515	0.01495	0.00029
1 day IL15 vs 180 days IL15	0.82403	1	1.94E-06
1 day IL15 vs 180 days SLURP1	0.00002	0	0
1 day IL15 vs 180 days TGFB1	0	0	0.07296
1 day IL15 vs 180 days TGFB2	0	0	0
1 day IL15 vs 180 days TGFB3	0	0	0

1 day IL15 vs 180 days VEGFA	0	0	0
1 day IL15 vs 30 days IHNB	0.27178	1	0.00021
1 day IL15 vs 30 days IL15	0.99285	1	1
1 day IL15 vs 30 days IL1B	9.86E-12	0	0
1 day IL15 vs 30 days SLURP1	0.00224	0.00162	0
1 day IL15 vs 30 days TGFB1	0	0	1.42E-08
1 day IL15 vs 30 days TGFB2	0	0	0
1 day IL15 vs 30 days TGFB3	0	0	0
1 day IL15 vs 30 days VEGFA	0	0	0
1 day IL15 vs 7 days IHNB	0.96453	0	9.98E-06
1 day IL15 vs 7 days IL15	1	0	1
1 day IL15 vs 7 days IL1B	0	0	0
1 day IL15 vs 7 days SLURP1	0.09589	0	0
1 day IL15 vs 7 days TGFB1	0	0	0.00118
1 day IL15 vs 7 days TGFB2	0	0	0
1 day IL15 vs 7 days TGFB3	0	0	0
1 day IL15 vs 7 days VEGFA	0	0	0
1 day IL1B vs 1 day SLURP1	0	0	0
1 day IL1B vs 1 day TGFB1	0.99997	0	0
1 day IL1B vs 1 day TGFB2	1.48E-06	0	0
1 day IL1B vs 1 day TGFB3	0.00229	0	0.0567
1 day IL1B vs 1 day VEGFA	0	1	0
1 day IL1B vs 180 days	2.85E-07	1	1
1 day IL1B vs 180 days IHNB	0	0	0
1 day IL1B vs 180 days IL15	0	0	0
1 day IL1B vs 180 days SLURP1	0	0	0
1 day IL1B vs 180 days TGFB1	0	2.29E-07	0
1 day IL1B vs 180 days TGFB2	0	1	0
1 day IL1B vs 180 days TGFB3	0	0.00036	2.63E-08
1 day IL1B vs 180 days VEGFA	1	0.78908	1
1 day IL1B vs 30 days IHNB	0	0	0
1 day IL1B vs 30 days IL15	0	0	0
1 day IL1B vs 30 days IL1B	0	1	1
1 day IL1B vs 30 days SLURP1	0	0	0
1 day IL1B vs 30 days TGFB1	6.24E-10	0.00006	0
1 day IL1B vs 30 days TGFB2	0.10431	0	0
1 day IL1B vs 30 days TGFB3	1.62E-11	1	5.12E-07
1 day IL1B vs 30 days VEGFA	0	0.99999	1
1 day IL1B vs 7 days IHNB	0	0.99999	0
1 day IL1B vs 7 days IL15	0	4.27E-07	0
1 day IL1B vs 7 days IL1B	0.99997	1	1
1 day IL1B vs 7 days SLURP1	0	3.31E-08	0

1 day IL1B vs 7 days TGFB1	1	0	0
1 day IL1B vs 7 days TGFB2	1	0	0
1 day IL1B vs 7 days TGFB3	0.99463	0.13605	2.28E-07
1 day IL1B vs 7 days VEGFA	0	1	0.00002
1 day SLURP1 vs 1 day TGFB1	0	0	0
1 day SLURP1 vs 1 day TGFB2	0	0	0
1 day SLURP1 vs 1 day TGFB3	0	0	0
1 day SLURP1 vs 1 day VEGFA	0	0	0
1 day SLURP1 vs 180 days	0	0	0
1 day SLURP1 vs 180 days IHNB	0	0	0
1 day SLURP1 vs 180 days IL15	0	0	0
1 day SLURP1 vs 180 days SLURP1	1	1	0
1 day SLURP1 vs 180 days TGFB1	0.56262	0.99982	0
1 day SLURP1 vs 180 days TGFB2	1.75E-06	0	0
1 day SLURP1 vs 180 days TGFB3	0.00107	0.92109	0
1 day SLURP1 vs 180 days VEGFA	0	0.00211	0
1 day SLURP1 vs 30 days IHNB	0	0	0
1 day SLURP1 vs 30 days IL15	1.30E-10	0	0
1 day SLURP1 vs 30 days IL1B	0.9709	1.02E-10	0
1 day SLURP1 vs 30 days SLURP1	1	0.00035	0
1 day SLURP1 vs 30 days TGFB1	0	0	0
1 day SLURP1 vs 30 days TGFB2	0	0	0
1 day SLURP1 vs 30 days TGFB3	0	1.93E-09	0
1 day SLURP1 vs 30 days VEGFA	0	1.01E-08	0
1 day SLURP1 vs 7 days IHNB	6.42E-12	0	0
1 day SLURP1 vs 7 days IL15	0.00013	0.99967	0
1 day SLURP1 vs 7 days IL1B	0	0	0
1 day SLURP1 vs 7 days SLURP1	1	0.99998	0
1 day SLURP1 vs 7 days TGFB1	0	0	0
1 day SLURP1 vs 7 days TGFB2	0	0	0
1 day SLURP1 vs 7 days TGFB3	0	0	0
1 day SLURP1 vs 7 days VEGFA	0	0	0
1 day TGFB1 vs 1 day TGFB2	0.42668	0	0.89555
1 day TGFB1 vs 1 day TGFB3	2.00E-12	0.86683	0
1 day TGFB1 vs 1 day VEGFA	0	0	0
1 day TGFB1 vs 180 days	0	0	0
1 day TGFB1 vs 180 days IHNB	0	0	0
1 day TGFB1 vs 180 days IL15	0	0	0
1 day TGFB1 vs 180 days SLURP1	0	0	0.69626
1 day TGFB1 vs 180 days TGFB1	0	0	1
1 day TGFB1 vs 180 days TGFB2	0	0	0.00066
1 day TGFB1 vs 180 days TGFB3	0	0	0

1 day TGFB1 vs 180 days VEGFA	0.86986	0	0
1 day TGFB1 vs 30 days IHNB	0	0	0
1 day TGFB1 vs 30 days IL15	0	0	0.00187
1 day TGFB1 vs 30 days IL1B	0	0	0
1 day TGFB1 vs 30 days SLURP1	0	0	0.13521
1 day TGFB1 vs 30 days TGFB1	0	0	1
1 day TGFB1 vs 30 days TGFB2	1.20E-08	0.9406	1.34E-09
1 day TGFB1 vs 30 days TGFB3	0	0	0
1 day TGFB1 vs 30 days VEGFA	0	0	0
1 day TGFB1 vs 7 days IHNB	0	0	0
1 day TGFB1 vs 7 days IL15	0	0	0.00004
1 day TGFB1 vs 7 days IL1B	1	0	0
1 day TGFB1 vs 7 days SLURP1	0	0	0.0166
1 day TGFB1 vs 7 days TGFB1	0.50519	0.23833	1
1 day TGFB1 vs 7 days TGFB2	0.59233	0.03365	1.16E-09
1 day TGFB1 vs 7 days TGFB3	1	0	0
1 day TGFB1 vs 7 days VEGFA	0	0	0
1 day TGFB2 vs 1 day TGFB3	0	3.25E-10	0
1 day TGFB2 vs 1 day VEGFA	0	0	0
1 day TGFB2 vs 180 days	0	0	0
1 day TGFB2 vs 180 days IHNB	0	0	0
1 day TGFB2 vs 180 days IL15	0	0	0
1 day TGFB2 vs 180 days SLURP1	0	0	1
1 day TGFB2 vs 180 days TGFB1	0	0	0.17368
1 day TGFB2 vs 180 days TGFB2	0	0	1
1 day TGFB2 vs 180 days TGFB3	0	0	0
1 day TGFB2 vs 180 days VEGFA	1.23E-11	0	0
1 day TGFB2 vs 30 days IHNB	0	0	0
1 day TGFB2 vs 30 days IL15	0	0	0
1 day TGFB2 vs 30 days IL1B	0	0	0
1 day TGFB2 vs 30 days SLURP1	0	0	1
1 day TGFB2 vs 30 days TGFB1	0	0	0.99997
1 day TGFB2 vs 30 days TGFB2	0	0	0.81428
1 day TGFB2 vs 30 days TGFB3	0	0	0
1 day TGFB2 vs 30 days VEGFA	0.03224	0	0
1 day TGFB2 vs 7 days IHNB	0	0	0
1 day TGFB2 vs 7 days IL15	0	0	0
1 day TGFB2 vs 7 days IL1B	0.42084	0	0
1 day TGFB2 vs 7 days SLURP1	0	0	1
1 day TGFB2 vs 7 days TGFB1	0	0	0.78102
1 day TGFB2 vs 7 days TGFB2	0	0.00014	0.80668
1 day TGFB2 vs 7 days TGFB3	0.84968	0	0

1 day TGFB2 vs 7 days VEGFA	0	0	0
1 day TGFB3 vs 1 day VEGFA	0	0	0
1 day TGFB3 vs 180 days	1	0	0.57291
1 day TGFB3 vs 180 days IHNB	0	0	0
1 day TGFB3 vs 180 days IL15	0	0	0
1 day TGFB3 vs 180 days SLURP1	0	0	0
1 day TGFB3 vs 180 days TGFB1	2.02E-06	0	0
1 day TGFB3 vs 180 days TGFB2	0.57658	0	0
1 day TGFB3 vs 180 days TGFB3	0.05671	0	1
1 day TGFB3 vs 180 days VEGFA	0.30231	0	0.99932
1 day TGFB3 vs 30 days IHNB	0	0	0
1 day TGFB3 vs 30 days IL15	0	0	0
1 day TGFB3 vs 30 days IL1B	1.31E-09	0	0.00099
1 day TGFB3 vs 30 days SLURP1	0	0	0
1 day TGFB3 vs 30 days TGFB1	1	0	0
1 day TGFB3 vs 30 days TGFB2	1	1.53E-08	0
1 day TGFB3 vs 30 days TGFB3	1	0	1
1 day TGFB3 vs 30 days VEGFA	0	0	0.87694
1 day TGFB3 vs 7 days IHNB	0	0	0
1 day TGFB3 vs 7 days IL15	0	0	0
1 day TGFB3 vs 7 days IL1B	2.17E-12	0	0.74245
1 day TGFB3 vs 7 days SLURP1	0	0	0
1 day TGFB3 vs 7 days TGFB1	0.71435	0	0
1 day TGFB3 vs 7 days TGFB2	0.63385	1	0
1 day TGFB3 vs 7 days TGFB3	0	0	1
1 day TGFB3 vs 7 days VEGFA	0	0	0
1 day VEGFA vs 180 days	0	1	0
1 day VEGFA vs 180 days IHNB	0	0	0
1 day VEGFA vs 180 days IL15	0	0	0
1 day VEGFA vs 180 days SLURP1	0	0	0
1 day VEGFA vs 180 days TGFB1	0	0	0
1 day VEGFA vs 180 days TGFB2	0	1	0
1 day VEGFA vs 180 days TGFB3	0	1.22E-09	0
1 day VEGFA vs 180 days VEGFA	0	0.01674	0
1 day VEGFA vs 30 days IHNB	0	0	0
1 day VEGFA vs 30 days IL15	0	0	0
1 day VEGFA vs 30 days IL1B	0	0.9728	0
1 day VEGFA vs 30 days SLURP1	0	0	0
1 day VEGFA vs 30 days TGFB1	0	0.13861	0
1 day VEGFA vs 30 days TGFB2	0	0	0
1 day VEGFA vs 30 days TGFB3	0	0.90585	0
1 day VEGFA vs 30 days VEGFA	0.36666	0.83267	0

1 day VEGFA vs 7 days IHNB	0	1	0
1 day VEGFA vs 7 days IL15	0	0	0
1 day VEGFA vs 7 days IL1B	0	0.99847	0
1 day VEGFA vs 7 days SLURP1	0	0	0
1 day VEGFA vs 7 days TGFB1	0	0	0
1 day VEGFA vs 7 days TGFB2	0	0	0
1 day VEGFA vs 7 days TGFB3	0	0.97542	0
1 day VEGFA vs 7 days VEGFA	1	1	0.27474
180 days vs 180 days IHNB	0	0	0
180 days vs 180 days IL15	0	0	0
180 days vs 180 days SLURP1	6.03E-11	0	0
180 days vs 180 days TGFB1	0.00796	0.00006	0
180 days vs 180 days TGFB2	0.99837	0.99949	0
180 days vs 180 days TGFB3	0.82004	0.01636	0.00005
180 days vs 180 days VEGFA	0.00202	0.98865	1
180 days vs 30 days IHNB	0	0	0
180 days vs 30 days IL15	0	0	0
180 days vs 30 days IL1B	0.00006	1	1
180 days vs 30 days SLURP1	0	0	0
180 days vs 30 days TGFB1	1	2.49E-07	0
180 days vs 30 days TGFB2	1	0	0
180 days vs 30 days TGFB3	1	1	0.00048
180 days vs 30 days VEGFA	0	1	1
180 days vs 7 days IHNB	0	0.99582	0
180 days vs 7 days IL15	0	0.0001	0
180 days vs 7 days IL1B	0	1	1
180 days vs 7 days SLURP1	0	0.00001	0
180 days vs 7 days TGFB1	0.02901	0	0
180 days vs 7 days TGFB2	0.018	0	0
180 days vs 7 days TGFB3	0	0.00853	0.00027
180 days vs 7 days VEGFA	0	1	9.20E-09
180 days IHNB vs 180 days IL15	1	0.34578	1
180 days IHNB vs 180 days SLURP1	0	0	0
180 days IHNB vs 180 days TGFB1	0	0	0
180 days IHNB vs 180 days TGFB2	0	0	0
180 days IHNB vs 180 days TGFB3	0	0	0
180 days IHNB vs 180 days VEGFA	0	0	0
180 days IHNB vs 30 days IHNB	1	0.82086	1
180 days IHNB vs 30 days IL15	1	2.08E-10	0.00003
180 days IHNB vs 30 days IL1B	0	0	0
180 days IHNB vs 30 days SLURP1	0	0	0
180 days IHNB vs 30 days TGFB1	0	0	0

180 days IHNB vs 30 days TGFB2	0	0	0
180 days IHNB vs 30 days TGFB3	0	0	0
180 days IHNB vs 30 days VEGFA	0	0	0
180 days IHNB vs 7 days IHNB	1	0	1
180 days IHNB vs 7 days IL15	0.99048	0	0.0018
180 days IHNB vs 7 days IL1B	0	0	0
180 days IHNB vs 7 days SLURP1	0	0	0
180 days IHNB vs 7 days TGFB1	0	0	0
180 days IHNB vs 7 days TGFB2	0	0	0
180 days IHNB vs 7 days TGFB3	0	0	0
180 days IHNB vs 7 days VEGFA	0	0	0
180 days IL15 vs 180 days SLURP1	0	0	0
180 days IL15 vs 180 days TGFB1	0	0	0
180 days IL15 vs 180 days TGFB2	0	0	0
180 days IL15 vs 180 days TGFB3	0	0	0
180 days IL15 vs 180 days VEGFA	0	0	0
180 days IL15 vs 30 days IHNB	1	1	1
180 days IL15 vs 30 days IL15	1	0.9879	1.34E-07
180 days IL15 vs 30 days IL1B	0	0	0
180 days IL15 vs 30 days SLURP1	0	2.25E-06	0
180 days IL15 vs 30 days TGFB1	0	0	0
180 days IL15 vs 30 days TGFB2	0	0	0
180 days IL15 vs 30 days TGFB3	0	0	0
180 days IL15 vs 30 days VEGFA	0	0	0
180 days IL15 vs 7 days IHNB	1	0	1
180 days IL15 vs 7 days IL15	1	0	0.00002
180 days IL15 vs 7 days IL1B	0	0	0
180 days IL15 vs 7 days SLURP1	0	0	0
180 days IL15 vs 7 days TGFB1	0	0	0
180 days IL15 vs 7 days TGFB2	0	0	0
180 days IL15 vs 7 days TGFB3	0	0	0
180 days IL15 vs 7 days VEGFA	0	0	0
180 days SLURP1 vs 180 days TGFB1	0.99999	0.99703	0.05263
180 days SLURP1 vs 180 days TGFB2	0.07144	0	1
180 days SLURP1 vs 180 days TGFB3	0.62577	0.75208	0
180 days SLURP1 vs 180 days VEGFA	0	0.00024	0
180 days SLURP1 vs 30 days IHNB	0	0	0
180 days SLURP1 vs 30 days IL15	0	0	0
180 days SLURP1 vs 30 days IL1B	1	2.10E-12	0
180 days SLURP1 vs 30 days SLURP1	1	0.00288	1
180 days SLURP1 vs 30 days TGFB1	3.74E-08	0	0.99923

180 days SLURP1 vs 30 days TGFB2	0	0	0.95059
180 days SLURP1 vs 30 days TGFB3	8.18E-07	4.95E-11	0
180 days SLURP1 vs 30 days VEGFA	0	2.95E-10	0
180 days SLURP1 vs 7 days IHNB	0	0	0
180 days SLURP1 vs 7 days IL15	2.02E-11	0.99519	0
180 days SLURP1 vs 7 days IL1B	0	0	0
180 days SLURP1 vs 7 days SLURP1	1	0.99941	1
180 days SLURP1 vs 7 days TGFB1	0	0	0.51933
180 days SLURP1 vs 7 days TGFB2	0	0	0.94754
180 days SLURP1 vs 7 days TGFB3	0	0	0
180 days SLURP1 vs 7 days VEGFA	0	0	0
180 days TGFB1 vs 180 days TGFB2	0.99985	0	1.59E-07
180 days TGFB1 vs 180 days TGFB3	1	1	0
180 days TGFB1 vs 180 days VEGFA	0	0.99431	0
180 days TGFB1 vs 30 days IHNB	0	0	0
180 days TGFB1 vs 30 days IL15	0	0	0.19334
180 days TGFB1 vs 30 days IL1B	1	0.05284	0
180 days TGFB1 vs 30 days SLURP1	0.99132	0	0.0009
180 days TGFB1 vs 30 days TGFB1	0.11868	0	1
180 days TGFB1 vs 30 days TGFB2	9.26E-10	0	0
180 days TGFB1 vs 30 days TGFB3	0.31072	0.13955	0
180 days TGFB1 vs 30 days VEGFA	0	0.22338	0
180 days TGFB1 vs 7 days IHNB	0	0	0
180 days TGFB1 vs 7 days IL15	0	1	0.02232
180 days TGFB1 vs 7 days IL1B	0	0.00672	0
180 days TGFB1 vs 7 days SLURP1	0.71555	1	0.00002
180 days TGFB1 vs 7 days TGFB1	0	0	1
180 days TGFB1 vs 7 days TGFB2	0	0	0
180 days TGFB1 vs 7 days TGFB3	0	0	0
180 days TGFB1 vs 7 days VEGFA	0	9.52E-09	0
180 days TGFB2 vs 180 days TGFB3	1	1.20E-12	0
180 days TGFB2 vs 180 days VEGFA	0	0.00052	0
180 days TGFB2 vs 30 days IHNB	0	0	0
180 days TGFB2 vs 30 days IL15	0	0	0
180 days TGFB2 vs 30 days IL1B	0.95596	0.71939	0
180 days TGFB2 vs 30 days SLURP1	0.00221	0	1
180 days TGFB2 vs 30 days TGFB1	1	0.56293	0.16872
180 days TGFB2 vs 30 days TGFB2	0.06949	0	1
180 days TGFB2 vs 30 days TGFB3	1	0.50398	0
180 days TGFB2 vs 30 days VEGFA	0	0.374	0
180 days TGFB2 vs 7 days IHNB	0	1	0
180 days TGFB2 vs 7 days IL15	0	0	0

180 days TGFB2 vs 7 days IL1B	0	0.939	0
180 days TGFB2 vs 7 days SLURP1	8.57E-06	0	1
180 days TGFB2 vs 7 days TGFB1	5.27E-12	0	0.00015
180 days TGFB2 vs 7 days TGFB2	1.60E-12	0	1
180 days TGFB2 vs 7 days TGFB3	0	0.99969	0
180 days TGFB2 vs 7 days VEGFA	0	1	0
180 days TGFB3 vs 180 days VEGFA	0	1	0.08969
180 days TGFB3 vs 30 days IHNB	0	0	0
180 days TGFB3 vs 30 days IL15	0	0	0
180 days TGFB3 vs 30 days IL1B	0.99994	0.59497	5.05E-12
180 days TGFB3 vs 30 days SLURP1	0.12196	0	0
180 days TGFB3 vs 30 days TGFB1	0.99085	0	0
180 days TGFB3 vs 30 days TGFB2	0.00084	0	4.65E-08
180 days TGFB3 vs 30 days TGFB3	0.99929	0.79312	1
180 days TGFB3 vs 30 days VEGFA	0	0.87812	0.00119
180 days TGFB3 vs 7 days IHNB	0	0	0
180 days TGFB3 vs 7 days IL15	0	1	0
180 days TGFB3 vs 7 days IL1B	0	0.25074	0.00026
180 days TGFB3 vs 7 days SLURP1	0.00336	1	0
180 days TGFB3 vs 7 days TGFB1	0	0	0
180 days TGFB3 vs 7 days TGFB2	0	0	5.27E-08
180 days TGFB3 vs 7 days TGFB3	0	0	1
180 days TGFB3 vs 7 days VEGFA	0	0.00003	0
180 days VEGFA vs 30 days IHNB	0	0	0
180 days VEGFA vs 30 days IL15	0	0	0
180 days VEGFA vs 30 days IL1B	0	1	0.99525
180 days VEGFA vs 30 days SLURP1	0	0	0
180 days VEGFA vs 30 days TGFB1	0.00003	0	0
180 days VEGFA vs 30 days TGFB2	0.89144	0	0
180 days VEGFA vs 30 days TGFB3	1.83E-06	1	0.23741
180 days VEGFA vs 30 days VEGFA	0	1	1
180 days VEGFA vs 7 days IHNB	0	0.00008	0
180 days VEGFA vs 7 days IL15	0	0.99645	0
180 days VEGFA vs 7 days IL1B	0.8732	0.99998	1
180 days VEGFA vs 7 days SLURP1	0	0.98021	0
180 days VEGFA vs 7 days TGFB1	1	0	0
180 days VEGFA vs 7 days TGFB2	1	0	0
180 days VEGFA vs 7 days TGFB3	0.46041	0	0.18606
180 days VEGFA vs 7 days VEGFA	0	0.54259	0
30 days IHNB vs 30 days IL15	1	0.804	0.00002
30 days IHNB vs 30 days IL1B	0	0	0
30 days IHNB vs 30 days SLURP1	0	6.28E-09	0

30 days IHNB vs 30 days TGFB1	0	0	0
30 days IHNB vs 30 days TGFB2	0	0	0
30 days IHNB vs 30 days TGFB3	0	0	0
30 days IHNB vs 30 days VEGFA	0	0	0
30 days IHNB vs 7 days IHNB	1	0	1
30 days IHNB vs 7 days IL15	0.99474	0	0.00137
30 days IHNB vs 7 days IL1B	0	0	0
30 days IHNB vs 7 days SLURP1	0	0	0
30 days IHNB vs 7 days TGFB1	0	0	0
30 days IHNB vs 7 days TGFB2	0	0	0
30 days IHNB vs 7 days TGFB3	0	0	0
30 days IHNB vs 7 days VEGFA	0	0	0
30 days IL15 vs 30 days IL1B	0	0	0
30 days IL15 vs 30 days SLURP1	0	0.94804	0
30 days IL15 vs 30 days TGFB1	0	0	2.45E-07
30 days IL15 vs 30 days TGFB2	0	0	0
30 days IL15 vs 30 days TGFB3	0	0	0
30 days IL15 vs 30 days VEGFA	0	0	0
30 days IL15 vs 7 days IHNB	1	0	8.07E-07
30 days IL15 vs 7 days IL15	1	0	1
30 days IL15 vs 7 days IL1B	0	0	0
30 days IL15 vs 7 days SLURP1	1.59E-11	0	0
30 days IL15 vs 7 days TGFB1	0	0	0.00654
30 days IL15 vs 7 days TGFB2	0	0	0
30 days IL15 vs 7 days TGFB3	0	0	0
30 days IL15 vs 7 days VEGFA	0	0	0
30 days IL1B vs 30 days SLURP1	0.99999	0	0
30 days IL1B vs 30 days TGFB1	0.00346	0	0
30 days IL1B vs 30 days TGFB2	0	0	0
30 days IL1B vs 30 days TGFB3	0.01997	1	1.83E-10
30 days IL1B vs 30 days VEGFA	0	1	1
30 days IL1B vs 7 days IHNB	0	0.50674	0
30 days IL1B vs 7 days IL15	0	0.06881	0
30 days IL1B vs 7 days IL1B	0	1	1
30 days IL1B vs 7 days SLURP1	0.99053	0.02206	0
30 days IL1B vs 7 days TGFB1	0	0	0
30 days IL1B vs 7 days TGFB2	0	0	0
30 days IL1B vs 7 days TGFB3	0	2.39E-06	6.85E-11
30 days IL1B vs 7 days VEGFA	0	1	0.00456
30 days SLURP1 vs 30 days TGFB1	2.29E-11	0	0.91048
30 days SLURP1 vs 30 days TGFB2	0	0	0.99978
30 days SLURP1 vs 30 days TGFB3	8.60E-10	0	0

30 days SLURP1 vs 30 days VEGFA	0	0	0
30 days SLURP1 vs 7 days IHNB	0	0	0
30 days SLURP1 vs 7 days IL15	3.36E-08	0	0
30 days SLURP1 vs 7 days IL1B	0	0	0
30 days SLURP1 vs 7 days SLURP1	1	0	1
30 days SLURP1 vs 7 days TGFB1	0	0	0.06189
30 days SLURP1 vs 7 days TGFB2	0	0	0.99975
30 days SLURP1 vs 7 days TGFB3	0	0	0
30 days SLURP1 vs 7 days VEGFA	0	0	0
30 days TGFB1 vs 30 days TGFB2	0.99938	0	0.00005
30 days TGFB1 vs 30 days TGFB3	1	0	0
30 days TGFB1 vs 30 days VEGFA	0	0	0
30 days TGFB1 vs 7 days IHNB	0	0.7661	0
30 days TGFB1 vs 7 days IL15	0	0	7.46E-10
30 days TGFB1 vs 7 days IL1B	0	1.29E-10	0
30 days TGFB1 vs 7 days SLURP1	0	0	0.58456
30 days TGFB1 vs 7 days TGFB1	0.001	0	1
30 days TGFB1 vs 7 days TGFB2	0.00051	0	0.00005
30 days TGFB1 vs 7 days TGFB3	0	1	0
30 days TGFB1 vs 7 days VEGFA	0	0.00061	0
30 days TGFB2 vs 30 days TGFB3	0.99171	0	1.94E-09
30 days TGFB2 vs 30 days VEGFA	0	0	0
30 days TGFB2 vs 7 days IHNB	0	0	0
30 days TGFB2 vs 7 days IL15	0	0	0
30 days TGFB2 vs 7 days IL1B	1.29E-08	0	0
30 days TGFB2 vs 7 days SLURP1	0	0	1
30 days TGFB2 vs 7 days TGFB1	0.99232	1	1.21E-10
30 days TGFB2 vs 7 days TGFB2	0.98523	0	1
30 days TGFB2 vs 7 days TGFB3	3.31E-11	0	4.86E-09
30 days TGFB2 vs 7 days VEGFA	0	0	0
30 days TGFB3 vs 30 days VEGFA	0	1	0.00725
30 days TGFB3 vs 7 days IHNB	0	0.29424	0
30 days TGFB3 vs 7 days IL15	0	0.17193	0
30 days TGFB3 vs 7 days IL1B	0	1	0.00193
30 days TGFB3 vs 7 days SLURP1	0	0.06881	0
30 days TGFB3 vs 7 days TGFB1	0.0001	0	0
30 days TGFB3 vs 7 days TGFB2	0.00005	0	2.23E-09
30 days TGFB3 vs 7 days TGFB3	0	2.13E-07	1
30 days TGFB3 vs 7 days VEGFA	0	0.99996	0
30 days VEGFA vs 7 days IHNB	0	0.19346	0
30 days VEGFA vs 7 days IL15	0	0.26651	0
30 days VEGFA vs 7 days IL1B	0	1	1

30 days VEGFA vs 7 days SLURP1	0	0.12144	0
30 days VEGFA vs 7 days TGFB1	0	0	0
30 days VEGFA vs 7 days TGFB2	0	0	0
30 days VEGFA vs 7 days TGFB3	0	4.65E-08	0.00449
30 days VEGFA vs 7 days VEGFA	0.80034	0.99981	7.05E-11
7 days IHNB vs 7 days IL15	1	0	0.00009
7 days IHNB vs 7 days IL1B	0	0.83071	0
7 days IHNB vs 7 days SLURP1	0	0	0
7 days IHNB vs 7 days TGFB1	0	0	0
7 days IHNB vs 7 days TGFB2	0	0	0
7 days IHNB vs 7 days TGFB3	0	0.99998	0
7 days IHNB vs 7 days VEGFA	0	1	0
7 days IL15 vs 7 days IL1B	0	0.00958	0
7 days IL15 vs 7 days SLURP1	0.00003	1	0
7 days IL15 vs 7 days TGFB1	0	0	0.00018
7 days IL15 vs 7 days TGFB2	0	0	0
7 days IL15 vs 7 days TGFB3	0	0	0
7 days IL15 vs 7 days VEGFA	0	1.88E-08	0
7 days IL1B vs 7 days SLURP1	0	0.00214	0
7 days IL1B vs 7 days TGFB1	0.5112	0	0
7 days IL1B vs 7 days TGFB2	0.59823	0	0
7 days IL1B vs 7 days TGFB3	1	0.00008	0.00113
7 days IL1B vs 7 days VEGFA	0	1	9.28E-10
7 days SLURP1 vs 7 days TGFB1	0	0	0.00535
7 days SLURP1 vs 7 days TGFB2	0	0	1
7 days SLURP1 vs 7 days TGFB3	0	0	0
7 days SLURP1 vs 7 days VEGFA	0	1.16E-09	0
7 days TGFB1 vs 7 days TGFB2	1	0	1.05E-10
7 days TGFB1 vs 7 days TGFB3	0.1247	0	0
7 days TGFB1 vs 7 days VEGFA	0	0	0
7 days TGFB2 vs 7 days TGFB3	0.17297	0	5.55E-09
7 days TGFB2 vs 7 days VEGFA	0	0	0
7 days TGFB3 vs 7 days VEGFA	0	0.33146	0

TGF-β1-3, transforming growth factor beta 1-3; *IL-1B-* interleukina 1 beta; IL-15, interleukin 15; IHNBA, inhibin beta A chain; VEGG, vascular endothelial growth factor A; SLURP1, secreted Ly-6_uPAR-related protein 1; PRK, photorefractive keratectomy;
FS-LASIK, femtosecond-assisted laser in-situ keratomileusis; SMILE, refractive lenticule extraction with small incision lenticule extraction
