# Supplementary material for: Comparative Analysis of Corneal Wound Healing: Differential Molecular Responses in Tears Following PRK, FS-LASIK, and SMILE Procedures
Source: Biomedicines. 2024 Oct 9;12(10):2289. doi: 10.3390/biomedicines12102289 (PMC11505177; doi:10.3390/biomedicines12102289)
Supplement: Supplementary file 1 [file biomedicines-12-02289-s001.zip › Table S3.pdf]

Table S3: Comparison of IL-1β, IL-15, and SLURP1 concentrations in patients before and after PRK, FS-LASIK, and SMILE based on participants' gender

LVC method	Cytokine [pg/mL]		IL-1 β					IL-15					SLURP1				
	Time		0 day	1 day	7 days	30 days	180 days	0 day	1 day	7 days	30 days	180 days	0 day	1 day	7 days	30 days	180 days
PRK	Mean± standard deviation	Female	63.57± 4.16	70.60 ±4.19	76.66 ±6.12	39.42 ±4.79	53.66 ±2.98	12.30 ±5.62	20.72 ±3.37	16.19 ±5.15	12.56 ±5.40	11.47 ±4.36	34.86 ±4.33	33.25 ±3.39	31.46 ±5.57	33.18 ±4.97	36.18 ±4.79
	Mean± standard deviation	Male	66.42± 3.98±	70.45 ±5.03	74.72 ±5.62	39.14 ±6.23	54.69 ±5.02	9.76± 3.80	20.02 ±5.19	17.78 ±5.68	13.84 ±7.42	11.57 ±4.24	34.85 ±5.27	29.24 ±5.63	32.48 ±7.52	34.72 ±4.30	34.97 ±3.44
	p-value		0.14	0.94	0.73	0.91	0.57	0.26	0.75	0.52	0.66	0.96	1.00	0.06	0.73	0.47	0.54
FS-LASIK	Mean± standard deviation	Female	60.59± 4.80	58.63 ±5.50	56.81 ±5.34	56.08 ±3.97	57.99 ±4.85	12.01 ±5.23	27.20 ±5.23	48.86 ±6.07	30.01 ±4.96	25.47 ±4.66	33.94 ±5.12	44.04 ±4.41	49.00 ±4.62	35.95 ±4.65	43.65 ±5.00
	Mean± standard deviation	Male	60.15± 5.05	58.72 ±4.35	55.98 ±4.86	55.46 ±6.68	56.97 ±7.77	10.50 ±5.06	26.73 ±4.36	47.71 ±5.31	31.34 ±4.98	26.20 ±4.81	34.05 ±5.27	45.41 ±4.82	46.13 ±4.54	34.81 ±4.04	44.65 ±3.42
	p-value		0.77	0.95	0.60	0.69	0.57	0.34	0.76	0.52	0.38	0.61	0.94	0.32	0.04	0.40	0.47
SMILE	Mean± standard deviation	Female	63.25± 5,34	63.05 ±6.63	61.54 ±5.31	63.38 ±5.62	61.36 ±4.60	11.54 ±5.61	22.18 ±5.21	22.01 ±4.74	22.81 ±5.14	12.29 ±3.80	36.27 ±5.57	96.34 ±5.25	37.98 ±4.40	38.36 ±4.08	36.42 ±5.06
	Standard dev.		5.34	6.36	5.31	5.62	4.60	5.61	5.21	4.74	5.14	3.80	5.57	5.25	4.40	4.08	5.06
	Mean± standard deviation	Male	59.84± 6.00	61.49 ±5.01	59.55 ±4.99	65.05 ±4.62	61.34 ±4.57	11.28 ±4.73	22.32 ±2.72	21.18 ±3.99	22.22 ±7.81	13.00 ±4.74	34.04 ±3.95	95.97 ±4.69	41.00 ±5.60	36.92 ±5.20	37.99 ±5.11
	p-value		0.06	0.43	0.24	0.34	0.99	0.88	0.92	0.57	0.76	0.59	0.19	0.82	0.05	0.31	0.34

IL-1β- interleukina 1 beta; IL-15, interleukin 15; SLURP1, secreted Ly-6_uPAR-related protein 1; PRK, photorefractive keratectomy; FS-LASIK, femtosecond-assisted laser in-situ keratomileusis; SMILE, small incision lenticule extraction
